# The Carotid Body as Part of a Unified Sympathoadrenal System of Neural Crest Derivatives: Insights from Two Centuries of Research

**DOI:** 10.3390/ijms262211129

**Published:** 2025-11-18

**Authors:** Dmitry Otlyga, Ekaterina Otlyga, Olga Junemann, Yuliya Krivova, Sergey Saveliev

**Affiliations:** Avtsyn Research Institute of Human Morphology of Federal State Budgetary Scientific Institution “Petrovsky National Research Centre of Surgery”, 117418 Moscow, Russia; tsvetkovakatya@mail.ru (E.O.); junemann@outlook.com (O.J.); homulkina@rambler.ru (Y.K.); embrains@mail.ru (S.S.)

**Keywords:** chromaffin tissue, paraganglia, sympathoadrenal system, neural crest derivatives, neuroendocrine system

## Abstract

The carotid body—a chemoreceptive derivative of the neural crest located at the bifurcation of the carotid artery—has been studied for over 282 years. The history of research into this small but vital organ is full of unexpected turns and offers many valuable lessons. Initially considered part of a unified system of paraganglia performing the endocrine function, the carotid body was later reclassified and recognized as a chemosensory organ. This article highlights the key controversies encountered by past researchers. These contradictions though largely forgotten, remain unresolved. The aim of our article is to propose a unified model of the carotid body that integrates its endocrine and chemosensory structural aspects. As we show, the main problem in studying the carotid body was its isolated investigation, detached from other organs of the sympathoadrenal system. Only a comprehensive analysis of the carotid body with other components of this system has allowed researchers to form a more complete understanding of both the structure and function of these formations. Contrary to the prevailing view of the carotid body as the main peripheral chemoreceptor organ, it may also perform endocrine functions during certain periods of human ontogeny. It is these potential functions that may explain the presence of certain morphological structures in the carotid body, the significance of which has until recently remained a mystery.

## 1. Introduction

The neural crest is a remarkable cellular structure formed at the border of the neural plate, giving rise to a wide variety of derivatives with fundamentally diverse functions. Neural crest cells differentiate into peripheral neurons and glia, chromaffin cells of the adrenal medulla and paraganglia, melanocytes, chondrocytes, osteocytes, and several other cell types [1,2,3]. Some authors also suggest that neural crest cells may contribute to certain epithelial cells of the salivary and lacrimal glands [4].

Although the derivatives of the neural crest in the adult organism fulfill completely distinct functions, and their morphological and molecular features may at first glance appear only remotely related to a common origin, they nonetheless maintain morphogenetic connections with one another. Moreover, the establishment of their functions often proceeds through similar mechanisms.

In embryology, it is well established that the processes of morphogenesis, even of seemingly unrelated organs, do not occur in isolation, but rather closely interact and mutually influence one another within the framework of a single organism. Therefore, a deep understanding of the morphogenesis and functions of any neural crest derivative (or indeed of any other organ) requires not only the study of its molecular, histological, and anatomical characteristics, but also comparative analysis with its related organs and an evaluation of its place within the organism as a whole.

However, science cannot advance to this more comprehensive, “integrative” level of understanding morphogenesis and organ function without first examining the individual properties of an organ in isolation from the organism as a whole. Yet, in the course of such reductionist approaches, there is always the risk of misinterpreting the findings obtained. The history of research on the carotid body—a small chemoreceptive organ located at the bifurcation of the common carotid artery—provides a prime example of this.

The literature already contains a substantial number of high-quality, detailed reviews and monographs devoted to various aspects of the morphology and physiology of the carotid body [5,6,7,8,9,10,11,12,13,14]. The history of investigations into this small but critically important organ of the sympathoadrenal system is full of unexpected turns and offers many valuable lessons. It provides numerous examples of how the very same data can be interpreted in completely opposite ways, depending on the initial assumptions of the investigators.

In this article, we will demonstrate the contradictions encountered by researchers in the past. Discovered by anatomists, histologists, and physiologists of the 20th century, these contradictions, though largely forgotten, remain unresolved. Our objective was to develop a unified model that reconciles conflicting predecessor data regarding the carotid body’s endocrine and chemosensory features.

As we will show in this work, the primary issue in the study of the carotid body was its isolated investigation, detached from the other organs of the sympathoadrenal system. We, however, reinterpret the carotid body as an essential component of an integrated sympathoadrenal system, rather than merely an isolated chemoreceptor. We state that only a comprehensive analysis of the carotid body in conjunction with other components of the sympathoadrenal system enabled researchers to develop a more complete understanding of both the structure and function of these organs. It turned out that, contrary to the prevailing view of the carotid body as the main peripheral chemoreceptor organ, it may also perform endocrine functions during certain periods of human ontogeny. It is these potential functions that may explain the presence of certain morphological structures within the carotid body, which had remained a mystery until recently.

This review is structured in the following sections. The chapter *A Brief History of Carotid Body Research* provides a concise overview of the history of carotid body studies, from its discovery to the mid-20th century. It outlines the emergence of two opposing views of the carotid body: as a chemosensor and as an endocrine organ. The *Morphology of the Carotid Body* chapter details the structural features of the carotid body, ranging from its gross anatomy to its ultrastructure. In the chapter *Morphofunctional Theories of Carotid Body Function*, we provide a comparative review of the main theories, highlighting the contradictory interpretations put forward by different authors. The chapter *The Role of the Carotid Body in Disease Development and Pathological Conditions* briefly examines the main diseases in whose pathogenesis the carotid body is hypothesized to play a role. A critical analysis of the currently existing theories is provided. Finally, in the chapter *A New Theory of the Unified Sympathoadrenal System*, we synthesize both our own findings and the work of predecessors to formulate our view of the carotid body as a key organ within a unified sympathoadrenal system.

## 2. A Brief History of Carotid Body Research

### 2.1. The First Descriptions of the Carotid Body

The carotid body was first described by Hardovicus Taube in his 1743 dissertation *Dissertationem inauguralem de vera nervi intercostalis origine* under the name *ganglion minutuum* [15]. Later, in his well-known treatise *Elementa Physiologiae Corporis Humani*, his teacher, Alberto von Haller, mentioned the carotid body, referring to it as *ganglion exiguum* [16].

Further studies of the 18th and the first half of the 19th centuries, according to the excellent historical review by Zak and Lawson [7], were limited to describing the anatomy of the carotid body. Thus, between 1751 and 1755, Andersch described the carotid body, calling it *gangliolum intercaroticum*. In 1772, Neubauer also referred to this organ, naming it *ganglion parvum*, and in 1833 Mayer again described it as *ganglion intercaroticum*. Mayer further noted that the glossopharyngeal nerve participates in the innervation of the carotid body. In the same year, Valentin described the artery supplying the organ, while Svitzer concluded that it can be innervated exclusively by the glossopharyngeal nerve, without participation of sympathetic fibers [7].

From anatomical studies, researchers soon turned to the histological structure of the carotid body.

In 1862, Luschka interpreted the carotid body as an aggregation of glandular tubules and accordingly named it *glandula carotica*. And although Luschka attempted to use the injection technique to visualize the capillary bed of the carotid body in detail, he was not successful. Due to the organ’s abundant vascular supply, Luschka observed only a homogeneous red staining of the tissue. At the same time, Luschka emphasized the similarity between the carotid body and the adrenal gland [17]. A more refined vessel injection technique was employed by Arnold in 1865. In contrast, Arnold in 1865 argued that these “glandular tubules” were in fact tortuous blood vessels forming vascular glomeruli, and thus referred to the organ as *glomeruli arteriosi intercarotici* [18]. Thus, Arnold denied the similarity of the carotid body to the adrenal and other glands, considering it to be more of a vascular formation.

This contradiction divided researchers into two camps. However, the difficulty lay in the fact that the histological methods of that era could not definitively prove either position.

A significant milestone in the study of neuroendocrine organs came with the work of Henri Stilling and Alfred Kohn. Using tissue-staining methods with chromium salts, first discovered by Bertholdus Werner in 1857 and later described in more detail by Henle in 1865 [19], they observed cells that stained brown in the presence of chromium salts [20,21]. They demonstrated that some cells of the carotid body belonged to the chromaffin type. However, authors noted that chromaffin cells were rarely found in the human carotid body and often absent altogether.

The work of Alfred Kohn also became pivotal in history, as he not only provided a brilliant description of chromaffin cells but also advanced the concept of the paraganglion system (which included, in addition to the carotid body, the adrenal medulla, the organs of Zuckerkandl, and other paraganglia) as a unified entity.

At the beginning of the 20th century, in parallel with studies of the carotid body, numerous researchers investigated the neural regulation of cardiovascular and respiratory functions. In the 1920s, the German scientist Heinrich Hering demonstrated that electrical and mechanical stimulation of the carotid sinus produced bradycardia and lowered arterial pressure [22]. His studies laid the foundation for the modern paradigm that assigns the carotid body the role of a chemosensory organ.

In the history of carotid body research, particular importance is attached to the work of the Spanish histologist Fernando de Castro and the Belgian physiologist Corneille Jean François Heymans.

De Castro studied both the carotid body and the carotid sinus. He carefully investigated the nerve fibers of the carotid body, establishing that its principal innervation derives from a branch of the glossopharyngeal nerve. Additional fibers enter from the superior cervical sympathetic ganglion and, to a lesser extent, from the vagus nerve. Based on experiments involving transection of the glossopharyngeal nerve at different levels, de Castro concluded that the carotid body possesses primarily afferent innervation. Summarizing his findings, he proposed that the organ functions to detect qualitative changes in blood chemistry and exerts reflex influences on other organs. Furthermore, he hypothesized that the role of the carotid sinus is to measure pressure in the carotid arteries. He also argued that the classification of the carotid body as a paraganglia is incorrect [23,24,25].

Independently, Heymans carried out own investigations. In the 1920s and 1930s, Corneille Heymans and colleagues published results demonstrating the role of the carotid body in the sino-carotid reflex in response to changes in blood chemistry [26,27,28]. Corneille Jean François Heymans was awarded the Nobel Prize *“for the discovery of the role played by the sinus and aortic mechanisms in the regulation of respiration”* in 1938 [29].

In 1939, Comroe [30], in 1943, Watt [31], and in 1946, Gernandt [32], showed that stimulation of peripheral chemoreceptors in animals induces reflex hyperventilation and elevates arterial pressure. Researchers found that the carotid body primarily responds to anoxia, as exclusion of the carotid and aortic bodies from the reflex arc abolishes the organism’s response to oxygen deprivation. These studies also demonstrated that the carotid body reacts more strongly to changes in the partial pressure of oxygen than to alterations in the partial pressures of carbon dioxide.

In 1951, Daly and Schweitzer investigated the effect of carotid body activation on bronchial motility [33]. Later, Nadel and Widdicombe conducted similar experiments with modifications [34]. The two groups produced contradictory results: the first concluded that carotid body chemoreceptor activation caused bronchodilation, whereas carotid sinus baroreceptor stimulation produced bronchoconstriction; the second group reported the opposite. Although these discrepancies remain unresolved, the observed influence of the carotid body on respiration inspired surgeons to explore the possibility of treating bronchial asthma by targeting the carotid body.

The first to perform carotid body resection (glomectomy) for asthma was the Japanese surgeon Nakayama [35]. In December 1961, he presented this treatment, performed in 3900 patients. The initial results were striking, and soon many surgeons adopted Nakayama’s method, performing both unilateral and bilateral resections.

Unfortunately, the early optimism was soon replaced by disappointment. Follow-up revealed that the initial reduction in frequency and severity of asthma attacks was only temporary. In many patients, attacks recurred, and in rare cases became even more severe. Moreover, in some patients with bilateral glomectomy, the respiratory reflex to hypoxia was lost [36,37].

The published surgical results were inconsistent. On closer examination, only a small fraction of studies included placebo controls. Histological verification of excised tissues was often omitted, and when performed, a substantial proportion revealed absence of the carotid body in the resected material [36].

Despite conflicting data from physiologists and surgeons, the paradigm that the carotid body is primarily a vital peripheral chemoreceptor had become widely accepted. Since then, most researchers in histology, ultrastructure, and molecular mechanisms have interpreted their findings within this theoretical framework. By default, only the chemoreceptive function was attributed to the organ, while its evident similarities with other paraganglia were long neglected.

### 2.2. The Carotid Body as a Chemoreceptor Organ: A Key Morphofunctional Contradiction

Particular difficulties were associated with the interpretation of the structural features of the carotid body. As mentioned, Kohn and Stilling considered some cells of the carotid body to be chromaffin [20,21]. However, de Castro later rejected their chromaffin nature entirely [23,24,25], and Watzka even proposed reclassifying the carotid body as a non-chromaffin paraganglion [38].

In 1908, Gomez described the histology of the organ in greater detail, identifying two cell types—type I and type II [39]. L. L. de Kock made significant contributions by applying Holmes’ silver impregnation technique, providing a detailed description of the organ’s major cell types and the course of its nerve fibers [40,41]. De Kock emphasized the susceptibility of carotid body tissue to autolysis, which could markedly affect results. He also suggested a neural origin for type I cells [41], later refined by Gould, who demonstrated that these cells receive innervation [42].

By the mid-20th century, electron microscopy was increasingly applied in research. The higher resolution of this method deepened understanding of the organ’s structure. Numerous studies described the ultrastructure of carotid bodies in different animal species. By the 1970s, the characteristics of type I and type II cells had been elucidated, and the properties of intraorgan nerve fibers investigated. The prevailing theory proposed that type I cells are the primary chemosensitive elements, whereas type II cells are analogous to glial cells.

Yet this theory, still dominant today, was strongly contested by the renowned carotid body researcher Tim Biscoe. While accepting the supportive role of type II cells, he argued that type I cells are not chemosensory elements, insisting instead that the primary sensors are the free nerve endings abundantly distributed within the organ [8].

Even more skeptical of the chemosensory theory were two neuromorphologists—Tatiana Andreevna Grigorieva and Nina Alexandrovna Smitten. Using comparative anatomical and embryological approaches across a wide range of animal species, they examined the carotid body in the broader context of the sympathoadrenal, nervous, and cardiovascular systems.

Grigorieva concluded that all paraganglia, including the carotid body, are not sensory but endocrine organs, participating in vascular motor regulation through catecholamine secretion [43].

Smitten emphasized the common origin and similarity among paraganglia, including the carotid body. Studying the evolutionary development of neural crest-derived chromaffin cells, she demonstrated that paraganglionic chromaffin cells regress or lose their ability to stain with chromium salts in regions where vascular structures, such as the branchial arterial arches, undergo atrophy. According to her, the carotid body is associated with these reduced vascular remnants. Thus, in Smitten’s view, the carotid body and related organs are nothing more than *“traces of the phylogenetic past of chromaffin tissue”* [44].

According to Grigorieva and Smitten, the carotid body is best regarded as a tissue structure inherited by mammals from the evolutionary history of neural crest-derived chromaffin cells, which have lost their original endocrine functions and survive only as rudimentary remnants.

Karnauchow P. N. shared the views of Grigorieva and Smitten in rejecting the chemosensory role of the carotid body [45]. However, unlike them, he suggested that its cells may function as endocrine glands rather than evolutionary vestiges. In his comprehensive review, Karnauchow highlighted contradictions in the prevailing theory and contributed a pathologist’s perspective. Based on all available data, he argued that Kohn’s original endocrine theory was correct.

Although the works of Grigorieva, Smitten, and Karnauchow were persuasive, they failed to overturn the dominant paradigm of the sensory role of the carotid body. Reevaluations of the chemosensory theory even extended to later hypotheses of homology between carotid body cells and the innervated neuroepithelial cells of fish gills [46,47]—an idea that also provoked strong debate [48,49].

Thus, by the second half of the 20th century, carotid body researchers were divided into two unequal groups. The larger group regarded the carotid body as a chemosensory organ, with type I cells as its primary chemoreceptive elements. The smaller group rejected this view, contending that the carotid body is fundamentally an endocrine organ, with chemosensory function carried out instead by free nerve endings not directly associated with type I cells.

To better understand the essence of these contradictions, we shall now turn to a more detailed discussion of the anatomy, histology, and ultrastructure of the carotid body.

## 3. Morphology of the Carotid Body

### 3.1. Anatomy of the Carotid Body

#### 3.1.1. Shape, Size and Location

The carotid body is a small paired organ of gray or grayish-brown color located in the region of the carotid bifurcation [39,50]. It lies within richly vascularized connective tissue fibers, which in humans form a thin, poorly developed capsule [45]. At the lower pole of the carotid body, the connective tissue fibers become more organized and form a ligament anchoring the organ to the carotid artery—the Mayer’s ligament [7,45].

Most often, the organ is located directly at the angle formed by the division of the common carotid artery into its internal and external branches. However, the carotid body may also be situated on the external carotid artery or on the internal carotid artery near the carotid sinus. More rarely, it can be found on the ascending pharyngeal artery or on the common carotid artery itself [51,52] (Table 1).

A case of unilateral agenesis of the carotid body has also been reported in a 53-year-old woman with complete absence of the right common carotid artery [53]. In this case, despite careful dissection of the glossopharyngeal nerve, the Hering’s sinus nerve could not be identified, and the carotid sinus was absent. This observation suggests that the development of the carotid body is induced by a population of cells derived from the third branchial arch.

The carotid body is most frequently ovoid in shape. Less commonly, it appears bilobed, in which case it resembles a V-shape oriented with its base toward the feeding artery. Occasionally, duplication occurs, with two carotid bodies present on one side. In such cases, the two organs are usually located close together, and their combined weight equals that of a single carotid body [51,52]. Very rarely, a leaf-like form is observed [51] (Table 2). Some uncertainty surrounds the nodular form of the carotid body: it is thought to be associated with pathological processes such as hyperplasia, though it may also result from insufficiently careful dissection, leaving residual fat deposits on the surface [51,52].

The dimensions of the carotid body vary from 1.8 × 1.2 × 0.7 mm to 6.2 × 3.5 × 2.8 mm. Its mass ranges from 1.9 to 47.4 mg, with an average of 12.9 mg [54]. Some contradictions exist regarding age-related changes in size: while some authors report age-related atrophy [39], others claim enlargement with age [52].

This apparent contradiction is likely explained by hypertrophy of the organ in individuals suffering from chronic hypoxia, for example, due to high-altitude living or diseases associated with chronic cardiovascular and respiratory insufficiency. A positive correlation has also been reported between the mass of the carotid body and that of the left and right ventricles of the heart [52,54].

#### 3.1.2. Blood Supply

**Figure 1:**
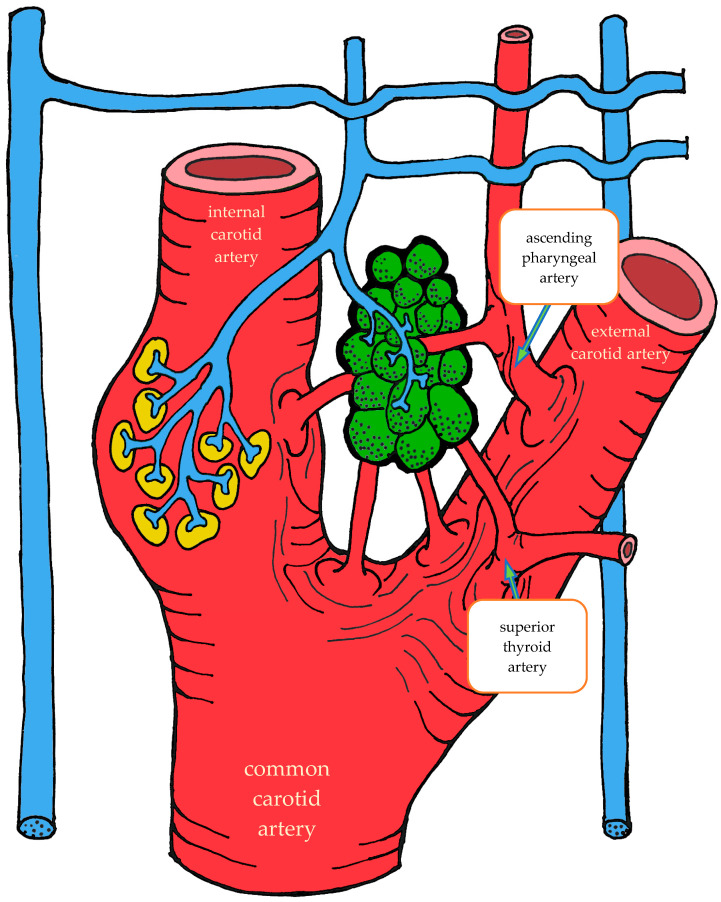
Variants of glomic artery origin according to Zak et al. [7], Heath et al. [55], and Jago et al. [56]. Nerves are shown in blue, carotid sinus baroreceptors in yellow, and the carotid body in green.

The carotid body is supplied by its own glomic artery, which runs within the aforementioned Mayer’s ligament. Both the position of the organ and the point of origin of the artery are variable. Most frequently, the artery arises from the bifurcation of the common carotid artery, though it may also originate from the external or internal carotid artery, the ascending pharyngeal artery, or the superior thyroid artery. In some cases, the carotid body is supplied by two or more arteries arising independently from these major vessels (Figure 1) [7,55,56].

A distinctive morphological feature of the glomic arteries is their structural type. Their length ranges from 1–2 to 3.5–4 mm, with a luminal diameter at the origin of 150–200 μm and a wall thickness of 25–60 μm [55,56]. The key feature of these vessels is that their wall is of the elastic type, whereas vessels of similar caliber elsewhere in the body are predominantly of the muscular type [55,56,57].

As the arteries approach the carotid body, they narrow in diameter. They may either enter the organ as a single trunk and branch internally into interlobular arteries [55], or branch externally into smaller trunks before entering the lower pole of the organ, subsequently subdividing into interlobular arteries [56].

The wall structure of the glomic arteries is not uniform throughout their course. It is composed mainly of circular bundles of elastic fibers of varying thickness, with a smaller contribution of collagen fibers, fibroblasts, and smooth muscle cells [55,56,57]. Proximally, the intima is separated from the media by a discontinuous elastic membrane, but closer to the carotid body the boundary between layers becomes indistinct [55]. The media also contains numerous unmyelinated nerve fibers, suggesting a possible baroreceptor function [55,56,57]. The intima is lined mainly by flattened endothelial cells [56].

After passing through the carotid body, blood is collected by venules that run between lobules in the connective septa. These venules radiate outward and drain into numerous small veins located in the capsule and surrounding connective tissue. Unlike the arteries, which branch in a tree-like pattern, the veins form a plexus that nearly encircles the carotid body [7,57].

At the upper pole of the organ, the venous plexus becomes more pronounced, giving rise to larger veins that drain the blood from the carotid body. These veins run through the intercarotid space and drain into the pharyngeal, superior laryngeal, and lingual veins [7].

In addition to direct capillary blood flow, numerous arteriovenous anastomoses have been identified in many mammals, located mainly at the periphery of the organ [58,59,60,61,62]. However, Donald Heath and colleagues found no such anastomoses in serial sections of the human carotid body [57].

#### 3.1.3. Innervation

Given the rather complex anatomy and topography of the nerves and their plexuses in the head and neck region, it is not surprising that numerous contradictions appear in the literature concerning the innervation of the carotid body. These discrepancies may be attributed to imperfect dissection techniques, investigator error, but most importantly to individual variability in the course of the nerves.

It is generally accepted that the glossopharyngeal nerve (cranial nerve IX) plays the principal role in the innervation of the carotid body. This is most often—but not always—mediated by its branch, the carotid sinus nerve, or sinus nerve (synonyms: Hering’s nerve, intercarotid nerve, de Castro’s nerve, descending branch of the glossopharyngeal nerve) [7,8,63,64,65,66]. The glossopharyngeal nerve emerges from the medulla oblongata by several rootlets in the groove posterior to the olive, above the vagus nerve, and together with the latter exits the skull through the jugular foramen. A few millimeters below the jugular foramen, the carotid sinus nerve branches from the glossopharyngeal nerve as one or two trunks, which subsequently fuse into a single stem.

However, Boyd noted that in some cases, after fusion, the nerve divides again into two or more trunks [67]. The sinus nerve runs parallel to the vagus nerve, close to the internal carotid artery. It may lie anteromedial, anterolateral, or anterior to the internal carotid artery [66]. In some instances, the sinus nerve may branch from the glossopharyngeal nerve inside the cranial cavity, or conversely, at a considerable distance (up to 4 cm) below the jugular foramen [68].

In its terminal part, the sinus nerve divides into multiple branches, some of which innervate the baroreceptors of the carotid sinus, while others enter the carotid body, most often at its upper portion [65,66]. Along its course, the sinus nerve gives off numerous communicating branches to adjacent nerves. These usually connect with the vagus nerve and the cervical sympathetic trunk, and more than one communicating branch may arise from the vagus nerve alone.

Communicating branches frequently originate from the pharyngeal branch of the vagus nerve or from its inferior ganglion; more rarely, they connect the sinus nerve with the superior cervical ganglion [66,68]. Thus, the terminal branches of the sinus nerve entering the carotid body may carry fibers not only from the glossopharyngeal nerve.

Although most studies support the dominant contribution of the sinus nerve to carotid body innervation, this may not always be the case. Based on 33 dissections, Sheehan and colleagues reported that the carotid body may also receive innervation from the intercarotid plexus, formed by branches of the glossopharyngeal and vagus nerves and the sympathetic trunk, while the sinus nerve may innervate only the carotid sinus without supplying the carotid body [68]. This anatomical variation was not confirmed by Gerard and Billingsley [65] or by Toorop [66], but their sample sizes were much smaller (2 and 12 subjects, respectively).

Additionally, nerve fibers from the superior cervical ganglion may directly reach the carotid body [65]. Svitzer, in 1863, also reported possible involvement of the hypoglossal nerve (cranial nerve XII) in carotid body innervation [69], although no subsequent studies have confirmed this [65,66,68].

Not all fibers approaching the carotid body necessarily innervate it. Some pass along the surface of the organ toward the lower pole without entering, while others traverse the organ without forming terminals and exit at the lower pole [65,68]. Both groups of fibers then enter Mayer’s ligament and continue to the carotid sinus or the external carotid artery [68].

For clarity, the aforementioned patterns of innervation are schematically depicted in Figure 2, Figure 3, Figure 4, Figure 5, Figure 6, Figure 7, Figure 8, Figure 9 and Figure 10.

All of the above highlights the considerable individual variability not only in the organ and its topography, but also in its vascularization and, in particular, its innervation. This understanding will prove important in interpreting the results of surgical studies on the carotid body.

### 3.2. Histological Organization

#### 3.2.1. Light Microscopy

In humans, the carotid body is enclosed by a thin capsule that is only weakly developed and closely associated with the surrounding connective tissue [45]. Some authors do not consider it a distinct capsule [14], since the adjacent connective tissue tends to proliferate with aging, making it difficult to clearly distinguish the capsule from the surrounding tissue.

From the capsule, connective tissue septa extend into the organ, carrying arteries, veins, and nerve fibers. These septa divide the organ into lobules [14], which are round or oval in shape and up to 565 μm in diameter [52]. Vessels and nerve endings enter the lobules via these septa [14].

The histoarchitecture of the organ changes with age. In embryos and fetuses, the carotid body contains minimal connective tissue [70], but with maturation, the amount increases [14]. In the elderly, lobules are separated by broader connective partitions, with some lobules undergoing atrophy and others displaying compensatory hypertrophy [14].

Each lobule consists of cells closely apposed to capillaries. These include two main types: chief cells, or type I cells, and supporting cells, or type II cells [14,54]. Type I cells, the principal parenchymal component, are polygonal in shape, with pale foamy cytoplasm and round nuclei containing 2–3 nucleoli. They are surrounded by type II cells [40,41,54], which are elongated and spindle-shaped, with nuclei containing more condensed chromatin compared to type I cells. With their processes, type II cells envelop clusters of 3–6 or more type I cells [7,14,54].

A cluster of type I cells surrounded by type II cells constitutes a functional unit of the organ, termed a glomerulus [7]. Numerous nerve fibers from the interlobular stroma run between the glomeruli, closely apposed to type I cells [40,41,42].

In addition to these cell types, ganglionic neurons, mast cells, and fibroblasts are also found in the carotid body, outside the lobules [14,71]. Studies in animals indicate that ganglionic neurons are unevenly distributed, being concentrated mainly where the sinus nerve enters at the rostral pole, with fewer neurons medially at the entry point of sympathetic fibers from the superior cervical ganglion [72].

Several authors distinguish three subtypes of type I cells in the human carotid body based on light microscopic features: light, dark, and pyknotic cells [11,52,54]. Some authors describe the pyknotic cells as progenitor cells and hypothesize that they contribute to organ growth and regeneration [14,73]. This classification is based on nuclear and cytoplasmic size as well as staining characteristics.

However, these subtypes have only been identified at the light microscopic level in humans [74]. Since early studies already noted the rapid loss of tinctorial properties of carotid body cells [41], it remains uncertain whether these morphological differences are genuine or artifacts of preparation. Researchers are divided on this issue.

Some argue that these subtypes are real, supported by electron microscopy demonstrating two subtypes of type I cells in both animals and humans [14]. Others, examining carotid bodies from patients with pulmonary and cardiac diseases [52,54] and from infants who died of sudden infant death syndrome [73], observed a correlation between disease presence and the ratio of dark to light cells.

Yet, ultrastructural differences do not always manifest at the light microscopic level. Thus, the second group of researchers maintains that the apparent subtypes observed with light microscopy result from autolytic changes and do not correspond to the genuine subtypes revealed by electron microscopy [74,75]. Our own findings support the latter view, suggesting that the observed differences are most likely artifacts of autolysis [76].

#### 3.2.2. Immunohistochemistry

Type I cells express a wide array of neuronal markers. Key among these are the cytoskeletal marker βIII-tubulin [49,77,78,79,80], PGP9.5 [49,80,81,82], and synaptophysin [49,80,82]. Some of these cells synthesize aromatic amino acid decarboxylase, dopamine β-hydroxylase [83], histidine decarboxylase [84], and tyrosine hydroxylase (TH) [49,77,78,80,84]. Notably, the percentage of TH-positive cells in humans is significantly lower than in rats and mice [77,84].

These cells also express several receptors, including dopamine D2 receptors [77,84], histamine H1 and H3 receptors [84], purinergic receptors (A2A and P2X2) [77,79], GABA receptors [77], and nicotinic acetylcholine receptors [77,79]. In addition, they possess two-pore domain potassium (TASK-1) channels and calcium-activated BK channels [77,79].

Type II cells synthesize proteins characteristic of glial populations, including glial fibrillary acidic protein (GFAP) [49,77,78,80], vimentin, nestin [77,78], and S100 [49,80,85]. Interestingly, type II cells also express the mechanosensitive proteins PIEZO1 and PIEZO2, although their precise role in the carotid body remains unclear [86].

Beyond the glomus cells themselves, the organ contains numerous nerve fibers expressing tyrosine hydroxylase, aromatic amino acid decarboxylase, dopamine β-hydroxylase, substance P, calcitonin gene-related peptide (CGRP), and neurofilaments of 160 kDa and 200 kDa [49,80,83,87].

Some of these fibers are branches of the glossopharyngeal nerve, while others originate from the superior cervical ganglion. According to Kummer and Habeck, significant immunohistochemical differences exist between fibers surrounding the lobules without entering them and those penetrating the lobules to form synaptic contacts with glomus cells [83,87].

Both types of fibers are immunopositive for tyrosine hydroxylase, but only those entering the lobules are immunopositive for 160 kDa neurofilaments. Conversely, these fibers are negative for aromatic amino acid decarboxylase and dopamine β-hydroxylase, which are instead present in fibers remaining in the interstitium without entering the lobules [83].

Nerve fibers containing substance P and CGRP are found both within the lobules and in the surrounding connective tissue. These fibers are immunonegative for tyrosine hydroxylase [87].

#### 3.2.3. Electron Microscopy

Most electron microscopic studies have been conducted in laboratory animals; however, the limited number of investigations performed on the human carotid body indicate that its principal ultrastructural features are similar to those in animals.

##### Type I Cells

Type I cells exhibit a rather complex morphology. Although at first glance they appear oval or polygonal, closer examination reveals processes extending up to three cell diameters from the soma. Microtubules with a diameter of about 200 Å can occasionally be found within these processes, while organelles and secretory granules are relatively rare [88].

The nuclei of type I cells are relatively large, rounded, and contain nucleoli. A defining ultrastructural feature is the presence of electron-dense secretory granules in the cytoplasm. In humans, these granules average 130 nm in diameter, although they range from 100 to 200 nm [88]. Their size varies among species—for example, 50–150 nm in cats [89,90] and 50–170 nm in rats [72]. Some authors report an even wider range, from 35 to 190 nm [91].

The Golgi apparatus is well developed, and secretory granules can be seen budding from its cisternae. The endoplasmic reticulum (ER) is also prominent, sometimes forming conspicuous parallel lamellae resembling Nissl substance [88].

Type I cells contain numerous mitochondria, 0.2–0.35 μm wide and up to 1.3 μm long. Branched mitochondria are frequently observed [91].

In the cytoplasm of some cells, irregular electron-dense inclusions ranging from 0.5 to 1 μm in diameter are present. Some, homogeneous and poorly demarcated, are thought to be lipid derivatives, while others, composed of dense clumps and lamellae with clear boundaries, represent lipofuscin deposits [88]. Adhesive junctions [91] and synapses [72,92] are observed between type I cells.

Thus, their morphology—characterized by electron-dense secretory granules, a well-developed Golgi apparatus, and extensive ER—supports the conclusion that type I cells are secretory in nature.

Electron microscopy has further revealed two subtypes of type I cells in both humans and animals: dark and light cells. This classification, however, remains controversial.

According to Grimley and Glenner, each lobule of the human carotid body contains 5–20% dark cells [88]. These cells have a more electron-dense cytoplasm, with a higher number of free or ER-associated ribosomes and a higher density of secretory granules compared to light cells.

Other authors, however, questioned the reliability of cytoplasmic density as a criterion, given the susceptibility to artifacts [72]. Moreover, Abbott and colleagues observed in cats that dark cells contain far fewer secretory granules than light cells [93], in direct contradiction to Grimley and Glenner’s findings [88].

Consequently, McDonald and Mitchell rejected classification based on cytoplasmic density. Instead, in their study of rat carotid bodies, they distinguished two types of type I cells—A and B—based on the diameter of secretory granules [72].

It should again be emphasized that ultrastructurally defined subtypes are not equivalent to those observed at the light microscopic level in humans. For example, McDonald and Mitchell described two ultrastructural subtypes in rat carotid bodies [72], although light microscopy reveals type I cells in this species to be largely homogeneous [74,76]. Furthermore, dark cells have been observed by light microscopy only in humans [74], whereas ultrastructural studies describe dark and light subtypes in a range of animal species as well as in humans [9,72,93].

##### Type II Cells

Type II cells possess long processes that greatly exceed the dimensions of the cell body. These processes taper with distance but do not branch. Where they contact one another, desmosome-like junctions are formed. With their processes, type II cells ensheath both nerve endings and type I cells [88]. However, this ensheathment is not complete: gaps are sometimes left, permitting direct contact between type I cells and the capillary basement membrane [91].

The nuclei of type II cells exhibit a more homogeneous chromatin distribution than those of type I cells. Depending on the plane of section, the nuclei appear oval, bean-shaped, or triangular. The cytoplasm contains a small number of simple or branched rough ER tubules; smooth ER is more prominent. Mitochondria are present but not abundant [88,91].

##### Ganglionic Neurons

Ganglionic neurons are found outside the lobules [91,94], surrounded by satellite glia. They possess large oval nuclei with conspicuous nucleoli and numerous nuclear pores. Their cytoplasm contains mitochondria and a well-developed Golgi complex. Rough ER cisternae form parallel lamellae resembling Nissl substance. Their surfaces bear numerous axodendritic and axosomatic synapses [94].

Nerve transection experiments demonstrate the presence of two types of ganglionic neurons in the carotid body: parasympathetic and sympathetic, with the former far more numerous. According to McDonald and Mitchell, the neuronal cell bodies innervating parasympathetic neurons of the organ are located in the brainstem, with their axons traveling via the glossopharyngeal nerve. Sympathetic ganglionic neurons, by contrast, receive innervation from neurons of the superior cervical ganglion [72].

##### Intralobular Nerve Endings

The carotid body possesses a rich innervation derived from the sinus nerve and the superior cervical ganglion. However, due to the organ’s complex architecture and the numerous processes of type I and type II cells, nerve fibers are not easily distinguished from surrounding cellular elements. They can, however, be identified by their regularly arranged microtubules (180–200 Å) and neurofilaments [88].

Nerve fibers are ensheathed by Schwann cells [88], but only a small proportion are myelinated; the majority are unmyelinated C-fibers [91]. Upon entering a lobule, they lose their Schwann cell covering and continue their course surrounded by type II cells [88]. Axons are separated from type I cells by type II cells, making contact only at synaptic sites [88], where they form varicosities 0.5–2 μm in diameter [91]. At these junctions, synaptic thickening of the membranes is observed [88].

Typically, a type I cell is contacted by a single nerve ending, though connections with two or three endings also occur [91]. Serial reconstruction studies by Kondo revealed that a single nerve fiber may innervate several type I cells [95].

According to Grimley and Glenner, most nerve varicosities in the human carotid body contain numerous synaptic vesicles 300–400 Å in diameter and many small mitochondria. Some vesicles also contain electron-dense granules [88]. Similar findings were reported by Biscoe and Stehbens in rabbits and cats [91]. Nishi, working on cats, distinguished two types of nerve endings on type I cells based on their form and size: large calyciform endings and small calyciform endings [96].

Large calyciform endings cover about 40% of the surface of type I cells. They resemble the endings described by earlier authors but contain fewer mitochondria. An interesting feature is the presence of finger-like projections penetrating into the type I cell body. However, no synaptic contacts between these projections and the cells were observed [96].

Small calyciform endings are smaller, contain fewer electron-dense granules, and tend to contact more than one type I cell [96].

McDonald and Mitchell further described bouton-shaped nerve endings. Using nerve transection experiments, they determined that endings originating from the superior cervical ganglion are bouton-shaped, whereas those from the sinus nerve may be either bouton-shaped or calyciform [72].

Based on these features, Grimley and Glenner concluded that such nerve varicosities represent sympathetic efferent endings. In their view, only a minority lacking abundant mitochondria and vesicles are likely afferent [88]. Biscoe expressed a similar view, suggesting that most endings on type I cells are motor rather than sensory [8]. Abbott likewise argued that the vesicles occasionally seen inside type I cells near synapses might simply be incidental [93].

Hervonen, studying human fetal carotid bodies, also agreed with Biscoe’s interpretation of an efferent character, noting that synaptic vesicles were consistently observed in the nerve endings [70]. Kobayashi, analyzing the ultrastructure of synapses in greater detail, found vesicles almost exclusively on the presynaptic side, leading him to classify the synapses morphologically as efferent [94]. King reported similar efferent features in the carotid bodies of birds [97].

Not all investigators concurred. Morgan reported that afferent synapses predominated in the rat carotid body, based on vesicle distribution and membrane features [92]. Kondo, using serial sections, found afferent synapses on type I cells to be 20 times more frequent [95].

By contrast, McDonald and Mitchell did not observe such a disparity. Using nerve transection, they designated endings from the glossopharyngeal nerve as “afferent” and those from the superior cervical ganglion as “efferent.” Approximately 95% of endings were “afferent” and 5% “efferent.” However, further analysis showed that 20% of “afferent” endings formed efferent synapses on type I cell bodies, 30% formed afferent synapses, 5% afferent synapses on type I processes, and another 5% reciprocal synapses with type I cells. About 40% made no synaptic contact with type I cells at all. All “efferent” endings studied formed efferent synapses on type I cells [72].

Thus, serious contradictions have arisen concerning the ultrastructure and function of synapses on type I cells. These inconsistencies may stem from artifacts introduced by different preparation methods, which greatly complicate interpretation. Kobayashi argued that the few vesicles observed at type I cell membranes are most likely artifacts [94], while Biscoe emphasized the profound impact of fixation methods on morphology [8].

Although conflicting, the bulk of evidence has favored the hypothesis that most endings on type I cells are sensory [7,14,64]. The strongest support comes from nerve transection experiments, which demonstrated no degeneration of endings on type I cells. However, it should be emphasized that the anatomical connection of an ending with a sensory neuron of the petrosal ganglion does not necessarily prove that it performs a sensory function. We will discuss this point in greater detail later.

In addition to the endings on type I cells, another distinct type of termination has been described: endings that terminate blindly within the processes of type II cells. These originate from unmyelinated fibers 0.3–3 μm in diameter, are fully ensheathed by type II cells, and contain many pleomorphic vesicles (1000–2000 Å), as well as small mitochondria and lamellar bodies. No synaptic contacts have been observed between these endings and surrounding type II cells, and their function remains unclear [96].

##### Stroma and Vessels

The organ is richly vascularized. Capillaries are relatively large, often exceeding 7 μm in diameter [91]. Endothelial cells lining them contain fenestrations 400–700 Å in diameter, a feature typical of endocrine organs. Endothelial cells rest on a continuous basement membrane, in whose clefts lie pericytes with cilia and processes. Beyond this lies interstitial tissue containing collagen fibers and fibroblasts. Larger vessels have a muscular coat. Mast cells are frequently found in the interstitium outside the lobules, near vessels [88].

Thus, between the capillary lumen and a type I cell lies a barrier consisting of endothelial cells, basement membrane with pericytes, stromal collagen, and type II cells.

##### Nerve Endings in the Stroma and Vessels

Numerous nerve fibers are also present in the stroma between lobules [72,83,87,88,91,96]. A notable feature is nerve endings directly contacting the capillary basement membrane. Along their course, these fibers are sheathed by Schwann cells, but at certain points the sheath is absent, leaving the ending in direct apposition to the basement membrane. These endings contain vesicles about 500 Å in diameter, as well as mitochondria and larger vesicles (650–1000 Å) with electron-dense cores [91].

Nerve transection experiments have shown that most of the unmyelinated fibers along vessels arise from the superior cervical ganglion [98]. Interestingly, although these fibers are postganglionic, they contain high levels of acetylcholinesterase and butyrylcholinesterase [99], suggesting that they may use acetylcholine as a neurotransmitter. McDonald and Mitchell found that after transection of only the sinus nerve, some fibers remained, whereas complete transection of all incoming nerves eliminated them [72]. These observations suggest that some fibers may in fact be axons of parasympathetic ganglionic neurons located within the carotid body.

## 4. Morphofunctional Theories of Carotid Body Function

From the preceding section, it becomes clear that the serious contradictions regarding the light and ultrastructural organization of the carotid body inevitably influenced the physiological interpretations of its role.

Initially, since the publication of Luschka’s work in 1862 [17], the prevailing view among scientists was that the carotid body possessed a secretory function. The studies of Kohn further reinforced this hypothesis. In his seminal monograph on paraganglia, Kohn concluded that the carotid body is one of the chromaffin paraganglia, whose function is catecholamine synthesis. He demonstrated its close association with the superior cervical ganglion and suggested its unity with the sympathoadrenal system [20].

Thus, the foundations of the endocrine theory of the carotid body were established.

However, not all investigators were able to detect catecholamines in the carotid body. Moreover, an endocrine organ would be expected to possess abundant efferent innervation. In the early 20th century, Fernando de Castro, employing nerve transection methods, showed that the principal source of innervation of the carotid body is afferent fibers of the glossopharyngeal nerve, rather than sympathetic fibers from the superior cervical ganglion. He also failed to detect significant catecholamine content in the organ [23,24,25]. Consequently, the studies of de Castro, supported by other researchers, delivered a major blow to Kohn’s theory of the endocrine function of the carotid body. At the same time, the work of Heymans on chemoreceptive zones firmly established the carotid body as a chemosensory organ.

In an attempt to resolve these contradictions, Watzka proposed dividing paraganglia into two groups: chromaffin, or sympathetic, and non-chromaffin, or parasympathetic [38]. According to his theory, sympathetic paraganglia are endocrine organs synthesizing catecholamines, while parasympathetic paraganglia—including the carotid body, aortic bodies, and several other head and neck paraganglia—are chemosensory organs responsible for detecting the partial pressures of blood gases.

Thus, the foundations of the chemosensory theory of the carotid body were laid, and this theory gained the widest acceptance.

The advent of electron microscopy and more refined biochemical methods further deepened our knowledge of the organ. It was discovered that de Castro’s observations were not entirely accurate: all type I cells do contain catecholamines, but in some animals—precisely those on which de Castro experimented—catecholamines are present only in low concentrations, below the detection threshold of earlier methods [44]. Nonetheless, the detection of catecholamines in the carotid body did not sway the proponents of the chemosensory theory.

It should be noted, however, that none of the supporters of the endocrine theory denied the existence of a chemoreceptor zone in the sino-carotid region, nor that this zone might reside within the carotid body. Their principal objection was to the identification of type I cells as the primary sensory elements.

Three hypotheses were advanced to explain the mechanism of chemoreception in the carotid body. The first, and most popular, proposed that type I cells are the primary sensory elements [64,77]. In contrast, the British histologist Tim Biscoe suggested that free nerve endings act as the sensors of blood gas partial pressures. A third hypothesis assigned this role to type II cells [8].

The third hypothesis was later rejected, since type II cells neither synthesize neurotransmitters, nor generate action potentials, nor form synaptic contacts with other elements of the organ [8].

For a long time, debate persisted between supporters of the first and second hypotheses. The main arguments in favor of the first included:

1. The absence of degeneration of nerve endings on type I cells following intracranial transection of the glossopharyngeal nerve (proximal to the ganglion), indicating an afferent role of these fibers [63,100].
2. Catecholamines contained in type I cell granules, regarded as neurotransmitters [7].
3. The ability of type I cells to alter their membrane potential in response to changes in the partial pressures of oxygen and carbon dioxide [101].
4. The release of catecholamines by type I cells in response to hypoxia [102].
5. Enlargement of the carotid body in high-altitude dwellers and patients with cardiovascular and pulmonary disease, due to proliferation of type I cells [54].
6. Restoration of hypoxia-induced impulses after reinnervation of the organ by a nerve normally lacking chemoreceptors [103].

However, Biscoe and colleagues revisited the experiments involving intracranial transection. Their work was distinctive in its extended observation period: animals were sacrificed 1.5 to 378 days post-surgery. By day 44, a statistically significant reduction in the number of nerve endings on type I cells was observed, along with signs of neurodegeneration [104].

Importantly, no changes in the electrophysiological activity of the sinus nerve were detected. This suggested that type I cells cannot be the primary chemosensory elements, and that their synapses represent efferent rather than afferent contacts. Biscoe hypothesized that the true chemosensors are the free nerve endings located in the stroma around vessels [8]. It can also be proposed that the endings terminating blindly within type II cell processes, without forming synapses with type I or type II cells, might also serve as chemosensors.

Later experiments involving transection of the glossopharyngeal nerve, however, failed to replicate the degeneration described by Biscoe. This discrepancy is likely explained by mechanical injury to the sensory ganglion during intracranial transection. In cats, the species used by Biscoe, the glossopharyngeal nerve possesses a ganglionic enlargement before entering the jugular foramen, making it particularly susceptible to trauma [96].

Thus, Biscoe’s findings are best explained as ganglion injury between the brainstem and the skull, which also accounts for the preservation of sinus nerve potentials: they were generated by intact neurons within the ganglion. Hence, Biscoe observed only a reduction, but not complete loss, of synapses on glomus cells.

Once Biscoe’s theory was refuted, discussion of chemoreception mechanisms proceeded entirely within the framework of the first hypothesis. Investigations focused narrowly on type I cells using physiological and molecular approaches, and all empirical data were interpreted through the lens of their chemosensory role.

Nevertheless, contradictions and intriguing details emerged. Pallot and Biscoe studied “wobbler” mutant mice, in which motor neurons degenerate with subsequent denervation of muscle cells, while sensory innervation of skin, mucosa, and Pacinian corpuscles remains intact. They found that the carotid body’s chemoreceptor response to changes in blood gas partial pressures was no different from that of healthy controls. Yet ultrastructural analysis revealed a striking reduction in innervation: only 0–7 normal endings per 100 type I cells in mutants, compared to 130–170 in controls. The authors suggested that this might reflect either redundant innervation or that synaptic contacts with type I cells are not essential for chemosensitivity [105].

Eyzaguirre, Baron, and Gallego showed that dissolved nitrogen increased sinus nerve activity without altering type I cell membrane potential. Acetylcholine and carbon dioxide acted similarly. However, when CO_2_ exposure lowered pH to 6, depolarization of type I cells was observed [106]. Acker and Pietruschka, studying carotid body cell cultures, found that decreasing oxygen partial pressure increased type I cell membrane potential, supporting their role as oxygen sensors [107].

Further studies revealed that carotid body cells contain large amounts of dopamine and small amounts of norepinephrine, and that hypoxia reduces dopamine content. This decrease is lessened by transection of the sinus nerve [108]. Hanbauer demonstrated that hypoxia increases tyrosine hydroxylase synthesis in type I cells [109].

Hellström confirmed the hypoxia-induced decrease in dopamine but found that dopamine inhibits sinus nerve activity [108]. Nishi likewise reported that dopamine, as well as dopamine receptor agonists such as apomorphine, suppress sinus nerve impulses [110]. By contrast, Zapata found that repeated administration of dopamine in small doses could actually increase activity [111]. Notably, norepinephrine did not affect sinus nerve function [112].

Thus, hypoxia induces both dopamine release from type I cells and increased sinus nerve activity, but dopamine itself inhibits signaling, at least initially. To reconcile this paradox, investigators suggested that acetylcholine may serve as the true mediator of transmission from type I cells to the sinus nerve. Indeed, acetylcholine has been shown to increase sinus nerve activity [106].

Christie reported that carotid body tumors induced hypotension in decapitated animals. However, this effect was not blocked by atropine, casting doubt on acetylcholine as the mediator [113]. Moreover, tumor tissue may synthesize substances not normally produced by the organ.

Fidone and colleagues, using gas chromatography and mass spectrometry, showed that transection of the sinus nerve increases acetylcholine levels in the organ [114]. However, Biscoe noted that large amounts of acetylcholine may also be present in postganglionic fibers from the superior cervical ganglion [99]. To resolve this, Fidone employed autoradiography, which revealed choline accumulation in type I cells of fully denervated carotid bodies, suggesting that these cells themselves synthesize acetylcholine [114].

Scientists have conducted numerous studies investigating the effects of various agonists and antagonists of cholinergic receptors. It was found that agonists increase sinus nerve activity, which is blocked by antagonists [115,116,117,118]. However, in experiments on cats, Douglas convincingly demonstrated that although the cholinergic receptor antagonist hexamethonium reduced sinus nerve activity in response to lobeline, nicotine, and acetylcholine, even at high doses it had no effect on the nerve’s response to hypoxia [119].

Eyzaguirre and Koyano repeated these studies on isolated carotid bodies. In a series of experiments, they showed that large doses of various cholinergic blockers do in fact reduce sinus nerve responses to hypoxia, but do not abolish them completely [115,120,121]. It should also be emphasized that, despite reduced activity, the nerve’s response to hypoxia was preserved to a greater extent than its response to acetylcholine. Furthermore, at such high doses of blocking agents, there is a significant risk of blocking conduction along the nerve fiber itself, which could explain the observed results [8].

For a long time, researchers were unable to directly demonstrate how hypoxia influences acetylcholine release. They were forced to rely on indirect methods, the results of which could be confounded by other mediators released by carotid body cells in addition to acetylcholine.

Direct evidence became available only in 2004, when Kim and colleagues showed that, unexpectedly, type I cells under hypoxia decreased acetylcholine release while increasing dopamine release [122]. Yet even here, contradictions arose: subsequent studies by Kåhlin and coauthors and by Fitzgerald and colleagues demonstrated that hypoxia actually increases acetylcholine release from type I cells [123,124]. Notably, Kim conducted his experiments on rabbit carotid bodies, Fitzgerald on cat and rat carotid bodies, and Kåhlin on human tissue. Whether these discrepancies reflect interspecies differences remains unresolved.

The next candidate for the role of transmitter mediating communication from type I cells to nerve endings was ATP. Among the first to describe the excitatory action of ATP on sinus nerve activity were the Soviet pharmacologists M. L. Belenky and S. V. Anichkov [118]. Investigating a wide range of substances, they found that ATP, like acetylcholine, excited carotid body nerves. Initially, however, the hypothesis that ATP served as a synaptic transmitter did not gain traction, as it was widely regarded as a nonspecific molecule [125].

Later, numerous authors confirmed the excitatory role of ATP in the chemoreceptor reflex. Fitzgerald [124] and Kåhlin [123] showed that during hypoxia type I cells release ATP. Thus, the hypothesis of ATP as a mediator transmitting signals from type I cells to sinus nerve endings gained broad acceptance.

Nevertheless, difficulties persisted. Experiments blocking purinergic receptors showed that these agents, like cholinergic antagonists, reduced sinus nerve responses to hypoxia but did not abolish them completely [125].

This led scientists to propose a new theory: that acetylcholine and ATP act together as co-transmitters. Yet even simultaneous blockade of cholinergic and purinergic receptors, with adequate doses of antagonists, reduced but did not abolish sinus nerve activity. The sinus nerve continued to respond to hypoxia and maintained basal activity [125,126].

It is now necessary to highlight several key experiments frequently cited by proponents of the primary role of type I cells in chemoreception.

The first was performed by Zapata, Hess, and Eyzaguirre in 1969 [103]. They transected the glossopharyngeal nerve and reinnervated its distal stump with the superior laryngeal nerve, which is thought to contain few motor fibers, many mechanoreceptors, and no intrinsic chemosensitivity to hypoxia, hypercapnia, or pH changes.

After 4–5 months, the researchers observed renewed impulses from the reinnervated carotid body in response to hypoxia. They proposed two possible explanations: (1) endings of the superior laryngeal nerve had changed their specificity to become chemosensitive; or (2) the endings retained their properties but were stimulated by substances released from type I cells.

The first explanation was rejected on the grounds that nerves cannot fundamentally change specificity. As an analogy, they cited motor fibers establishing contacts with sensory organs: the structural connections may form, but the endings are not functional. Therefore, their hypothesis was that acetylcholine released from type I cells in response to hypoxia was responsible for exciting superior laryngeal nerve fibers.

However, electron microscopic studies cast doubt on this hypothesis. Structural analysis revealed that synapses between type I cells and the newly sprouted endings were virtually absent. Instead, the endings penetrated into type II cells but did not reach type I cell membranes. Thus, no synapses were formed between presumed chemosensory cells and afferent endings; the two were separated by type II cell cytoplasm.

The researchers explained this by suggesting that acetylcholine might diffuse through the carotid body to reach the endings. Supporting their interpretation, they noted that the threshold for activation by natural stimuli was higher than in normal carotid bodies. In their view, if nerve endings were themselves primary sensors, the response should have matched controls.

Yet Monti-Bloch’s 1983 work showed that the superior laryngeal nerve does contain chemosensitive fibers, which respond to elevated CO_2_ partial pressure [127]. To overcome interpretive difficulties, Monti-Bloch, Stensaas, and Eyzaguirre conducted a second experiment: transplanting the carotid body into the skeletal muscle of cat hindlimbs, which lacks chemosensitive fibers. The grafts displayed chemosensitivity to stimuli such as hypoxia, hyperoxia, and hypercapnia. Ultrastructural analysis revealed new synapses on type I cells, although the published micrographs were of insufficient quality to evaluate synaptic morphology.

A third experiment was performed by Verna, Roumy, and Leitner, involving cryodestruction of carotid body cells. Following local freezing, both organ cells and nerve endings degenerated. After 18 days, regeneration of nerve trunks within the organ was observed, with restoration of baroreceptor activity but markedly diminished chemosensitivity: no responses to NaCN or oxygen partial pressure changes were detected [128].

Finally, Zhong, Zhang, and Nurse studied co-cultures of petrosal ganglion neurons with type I carotid body cells. They found that hypoxia altered the membrane potential only of type I cells, not neurons. However, when co-cultured, new contacts formed between neurons and clusters of type I cells, after which neurons displayed rapid depolarization and a surge in spike activity [129].

Thus, despite contradictions and unresolved issues, the theory of type I cells as the primary chemosensors has become generally accepted—a view ultimately acknowledged even by its staunch opponent, Biscoe [130].

Within this framework, multiple hypotheses emerged to explain the molecular mechanisms of cellular sensitivity to hypoxia, involving genes, RNA synthesis, and various signaling molecules [131,132,133,134,135,136,137]. The culmination of these studies was an integrated model of signal transmission from mitochondria to the membrane [5,101]. This model unifies numerous findings into a coherent theory of type I cell responses to hypoxia.

According to this theory, mitochondrial complex IV, which transfers electrons from cytochrome C to oxygen, plays the central role. During acute hypoxia, reduced oxygen supply to complex IV slows electron transfer, causing accumulation of reduced ubiquinone. Elevated ubiquinone levels in turn slow complex I activity, raising cytoplasmic NADH and reactive oxygen species concentrations. These molecules modulate ion channel activity in the cell membrane. The resulting alterations depolarize type I cells, leading to calcium influx and neurotransmitter release [5].

Although this theory elegantly explains intracellular mechanisms of chemosensitivity, in its current form it cannot resolve all outstanding contradictions. The presence of efferent synapses on endings of sensory nerves, the abundance of different neurotransmitters (including catecholamines) in type I cells, and the persistence of sinus nerve responses to hypoxia even after cholinergic and purinergic receptor blockade—all remain unexplained.

In our view, the principal limitation of interpreting the carotid body solely as a chemosensor is the neglect of its relationship with other elements of the sympathoadrenal system, such as the adrenal medulla, organs of Zuckerkandl, and other sympathetic paraganglia. Such a narrow interpretation both underestimates its likely endocrine functions and overestimates its role in various diseases.

Before presenting a new theory of carotid body function, we will briefly review the principal interpretations of the organ’s role in disease.

## 5. The Role of the Carotid Body in Disease Development and Pathological Conditions

Discussion of the carotid body’s influence on disease progression began almost immediately after its recognition as the principal peripheral chemosensor.

In this article, we will not address diseases directly associated with carotid body tissue itself (e.g., neoplasms, autoimmune glomitis), nor its potential roles as a metabolic sensor in diabetes mellitus [124,138] or as a regulator of immune functions [139,140]. Here, we limit our focus to its role in the development of cardiovascular and respiratory diseases.

### 5.1. Sudden Infant Death Syndrome (SIDS)

Considering the carotid body as the main peripheral chemoreceptor, several researchers have suggested a direct link between this organ and sudden infant death syndrome (SIDS).

Most studies on the carotid body in SIDS have focused either on changes in the volume and number of electron-dense granules or on shifts in the ratios of light, dark, and pyknotic (progenitor) cells.

Naeye and colleagues found that infants who died of SIDS had carotid bodies that were relatively smaller in proportion to total body size than those of infants who died from other causes. However, infants with SIDS who also had concomitant infectious pathology showed larger carotid bodies compared to fetuses without infections [141].

Cole and coauthors reported that in infants who died of SIDS, type I cells contained significantly fewer electron-dense granules, which in some cases were entirely absent. The chemoreceptor cells themselves were smaller in size compared to controls. The authors suggested that the lack of granules, which normally mediate synaptic transmission to nerve endings, could impair signaling [142].

In our view, this interpretation presents serious contradictions. Electron-dense granules are known to contain primarily catecholamines, including dopamine [143], which does not excite but rather inhibits sinus nerve activity. Thus, a reduction in electron-dense granules in cells could not plausibly lead to SIDS.

Perrin and colleagues repeated Cole’s study but concluded that changes in the carotid body—including the number and size of electron-dense granules—were not statistically significant [144].

Donald Heath and Paul Smith noted that until the 23rd week of development, carotid body cells appear monomorphic. After this stage, light, dark, and pyknotic (progenitor) subtypes of type I cells, as well as type II cells, become distinguishable. They also observed an increase in dark cells in infants who experienced hypoxia as a result of various illnesses [14].

Based on this classification, Pavai and coauthors examined the carotid bodies of infants who died from SIDS at the light microscopic level. Their morphometric analysis showed that carotid bodies of SIDS infants contained significantly more progenitor cells compared to controls [73].

However, as discussed earlier, classification of type I cells into three subtypes under light microscopy lacks convincing justification, as these categories are most likely artifacts of autolysis [74,75,76].

Lack and colleagues attempted a more comprehensive study on a large sample (89 infants), combining ultrastructural, light microscopic, and biochemical analyses. Their results showed no statistically significant differences between controls and SIDS infants [145].

It should also be noted that SIDS typically occurs at home rather than in a hospital setting, resulting in a longer interval between death and tissue fixation. By contrast, control carotid body samples are often obtained from hospital deaths, where the interval between death and fixation is usually shorter. Thus, morphological differences previously attributed to disease may actually reflect greater autolysis in SIDS cases.

In conclusion, there is currently no convincing data to suggest that the carotid body directly influences the course of SIDS.

### 5.2. Bronchial Asthma

Hypotheses that the carotid body may influence the course of bronchial asthma began to emerge following physiological studies investigating the organ’s role in bronchial motility.

In 1951, Daly and Schweitzer published a study showing that stimulation of carotid body chemoreceptors caused bronchodilation, whereas activation of carotid sinus baroreceptors produced bronchoconstriction [33]. In 1961, American physiologists Nadel and Widdicombe modified the experiment. Separately analyzing tracheal volume and pulmonary resistance, they concluded the opposite: activation of the carotid body caused bronchoconstriction, while stimulation of carotid sinus baroreceptors led to bronchodilation [34].

These contradictory results are difficult to reconcile. If the observed changes in bronchial motility were genuine rather than methodological artifacts, the discrepancy was most likely due to differences in anesthesia. The first group used nembutal, while the second used chlorasol, urethane, and morphine. This explanation is plausible, as Daly and Schweitzer themselves noted that chlorasol anesthesia reversed their results. Thus, the anesthesia likely affected not the carotid body itself but certain nuclei within the brainstem.

Surgical studies of carotid body resection in bronchial asthma patients also failed to resolve the contradictions [37,146,147]. Only a small fraction included placebo controls. Histological analysis of excised tissues was often omitted, and when performed, a substantial proportion of specimens did not contain carotid body tissue [36].

Gilevich and colleagues examined 100 carotid bodies from asthma patients and found that with disease duration exceeding 10 years, there was marked degeneration of parenchyma and proliferation of connective tissue. They concluded that the longer the disease persisted, the less parenchyma remained, and the more connective tissue proliferated. No clinical improvement was observed in patients who underwent bilateral glomectomy [148]. However, this study lacked instrumental assessments, controls, and microphotographs. The descriptions also raise doubts, since connective tissue proliferation is a normal feature of the organ.

Makarova and coauthors reported significant long-term improvement (3–6 years postoperatively) in patients [149]. However, they provided no histological data.

A more rigorous study was carried out by N. S. Koroleva. Among 131 glomectomies, short-term improvement was observed in 91% of patients. However, long-term outcomes were less favorable: 41% showed no reduction in medication dosage, 33% required higher doses, and only 15.2% were able to reduce their therapy. Histological analysis revealed carotid body tissue in only 61.8% of specimens; in 29.8% it was absent, and in 8.4% no histological examination was performed [150].

One of the largest clinical series was reported by E. S. Karashurov and colleagues. Summarizing 30 years of surgical experience with more than 3000 patients with severe or moderate asthma, they claimed that unilateral glomectomy produced sustained improvement in 69.5% of cases with follow-up exceeding two decades, with 23.2% achieving clinical remission [151].

Building on this, part of Karashurov’s team developed new surgical approaches for asthma. Rather than destroying the carotid body and other autonomic structures, they aimed to modulate their activity using implantable second- and third-generation neurostimulators targeting the sino-carotid nerve and sympathetic trunk. According to their results, this method not only reduced frequency and severity of attacks but also significantly decreased daily requirements for adrenergic and hormonal drugs [152].

However, despite these reported successes, both approaches suffer from a critical limitation: no blinded, placebo-controlled studies were performed, substantially weakening their evidential value. The authors did not clarify whether sham surgeries were conducted. This omission is particularly important, as earlier investigators had noted the strong influence of psychological factors, including placebo effects, on asthma outcomes. Blinded placebo-controlled studies accounting for this factor consistently found improvement in both experimental and control groups, without significant differences between them [153,154].

Thus, despite the large number of studies, there remains no convincing evidence to support surgical intervention on the carotid body for the treatment of bronchial asthma. Furthermore, the frequent absence of carotid body tissue in resected specimens underscores the need to carefully consider individual anatomical variability in future research.

### 5.3. Arterial Hypertension

Several authors have reported enlargement of the carotid body in individuals living at high altitudes, in patients with arterial hypertension, and in those with myocardial hypertrophy [54,155]. This led some researchers to hypothesize that the organ may contribute to elevated arterial pressure. They suggested that persistent hypertension is driven by increased basal activity of carotid body chemoreceptors [156,157].

However, this hypothesis is highly questionable, since there are currently no methods capable of distinguishing impulses from carotid sinus baroreceptors from those originating in the carotid body itself. The limited results of surgical operations in humans are, in our view, inconclusive [158,159]. Moreover, even if blood pressure and cardiac activity do normalize after carotid body removal, there is no definitive evidence that this is due to interruption of chemoreceptor activity rather than disruption of baroreceptor input.

## 6. A New Theory of the Unified Sympathoadrenal System

More than 282 years have passed since the discovery of the carotid body. Yet the question of its nature and function remains unresolved. Thanks to the experiments of de Castro and Heymans, the organ has been firmly established as a chemosensory structure, while the earlier hypothesis of an endocrine role has been discarded by most authors.

Since then, scientific efforts have focused on identifying the primary element responsible for the organ’s sensitivity to changes in the partial pressures of oxygen and carbon dioxide in the blood. The abundant innervation of type I cells by fibers originating from sensory neurons of the glossopharyngeal ganglion has led most researchers to favor the theory of their primary chemosensitivity. The objections of Abbott [93], King [97], Kobayashi [94], Hervonen [70], and Biscoe [8]—that most synapses on these cells are efferent—were largely ignored.

Later, Biscoe’s nerve transection experiment proved to be erroneous [72,160]. At present, it is an established fact—confirmed repeatedly by multiple methods—that nerve fibers innervating type I cells are axons of sensory neurons located in the glossopharyngeal ganglion.

The final doubts regarding the chemosensory role of type I cells were dispelled by experiments showing that chemosensitivity was abolished following destruction of the cells [128], and that isolated glossopharyngeal ganglion neurons in culture failed to respond to hypoxia unless they established contacts with type I cells [129].

Nevertheless, the currently accepted molecular model of signal transduction—from mitochondria to the membrane—cannot resolve several outstanding questions. What is the biological significance of the large number of efferent synapses on sensory nerve endings and of the wide variety of neurotransmitters, including catecholamines, in the cytoplasm of type I cells? Why does sinus nerve activity persist in response to hypoxia even after blockade of acetylcholine and purinergic receptors?

The first question can be reformulated as follows: does the carotid body possess a distinct endocrine function? If so, what is its biological significance?

The second question reduces to whether nerve endings themselves may act as direct oxygen sensors.

To address these issues, we conducted a series of studies in our laboratory on both antenatal and postnatal carotid bodies, investigating them in conjunction with the adrenal medulla and organs of Zuckerkandl. In this section, we summarize our previous findings alongside those of other authors.

### 6.1. Ontogenesis of the Human Carotid Body

Our data show that even at the earliest stages of development (8 weeks post-conception), the carotid body already constitutes a relatively large structure, with a cross-sectional area approximately seven times greater than that of the internal carotid artery. At this stage, the formation of glomeruli composed of two cell types is already evident. The presence of high levels of tyrosine hydroxylase suggests that the carotid body functions as an active organ from this period onward [49].

Our findings are consistent with those of Korkala and Hervonen, who, using formalin-induced fluorescence, also detected catecholamines in the cells of 8-week embryos [161]. However, their embryo had a crown-rump length of 28 mm, whereas the embryo we studied measured 24 mm, indicating an earlier developmental stage.

As gestation progressed, type I cell cytoplasm expanded and acquired weak eosinophilia. By 13–14 weeks, lobules formed by glomeruli were observable [49], somewhat earlier than reported by Scraggs and colleagues [162]. Thereafter, only further growth of the organ and an increase in the number of lobules were noted.

At all studied stages of prenatal development, we observed a high TH/βIII ratio, suggesting robust catecholamine synthesis throughout antenatal ontogenesis. As the organ grew, its relative size decreased. For example, at 8 weeks, the largest dimension of the organ was 3–4 times greater than the diameter of the internal carotid artery; by 10 weeks, it was 1.86 times larger; and in later stages, only 1.1–1.5 times larger. At the earliest stages, the close packing of cells made it difficult to trace nerve fibers within the organ. However, the presence of a well-developed neural network by 13–14 weeks suggests abundant innervation even earlier [49].

Examination of adult carotid bodies revealed further reduction in relative size. The maximal transverse dimension of the carotid body did not exceed one-third of the internal carotid artery’s diameter, averaging only about one-quarter. With age, there was marked proliferation of connective tissue and a corresponding decline in parenchyma, particularly in older individuals. This may indicate a diminished endocrine role of the organ in adults [49,80].

Comparisons between individuals revealed significant variability in the density of neurofilament-positive nerve fibers in carotid bodies, likely due to loss of type I cells while the neural apparatus remained intact. Notably, although we did not conduct physiological testing, most patients, according to medical histories, did not suffer from chronic respiratory insufficiency. Thus, despite pronounced organ atrophy in elderly patients, respiratory function was preserved sufficiently for normal daily activity [49,80].

These findings agree with Pokorski and colleagues, who observed no differences in hypoxia responses between young women (mean age 24) and older women (mean age 71) [163].

Our data further showed that the most striking difference in adult carotid bodies was a very low TH/βIII ratio in type I cells [49]. Literature indicates that even by 4 months of age, the proportion of tyrosine hydroxylase-positive cells is relatively low [84]. This distinction not only separates adult and pediatric organs from embryonic and fetal carotid bodies but also from those of rats, which remain rich in tyrosine hydroxylase, as confirmed by our data and that of other researchers [49,76,77,80,84].

However, this does not imply higher catecholamine content in rat type I cells. Despite producing abundant tyrosine hydroxylase, rat type I cells have very low catecholamine levels, indicating a slowdown of synthesis at later biochemical stages. Nonetheless, tyrosine hydroxylase production declines in rats within two days after birth [164]. Several authors note that while type I cell sensitivity is minimal at birth, it increases substantially with maturation [165,166,167].

### 6.2. The Carotid Body as Part of the Unified Sympathoadrenal System

When considered together with the organs of Zuckerkandl and the adrenal medulla, the carotid body presents an intriguing picture. During embryonic and fetal development, the human carotid body contains a large number of tyrosine hydroxylase-positive cells and is relatively large in size. The organs of Zuckerkandl undergo even greater development during the prenatal period. Our data therefore indicate that in the embryonic and fetal stages, the carotid body already represents a relatively massive structure, while at the same time the adrenal medulla contains only small, scattered groups of cells, most of which remain immature. This pattern suggests that during prenatal development, the carotid body performs an endocrine function alongside the organs of Zuckerkandl. Both organs synthesize catecholamines, thereby compensating for the functional immaturity of the adrenal medulla [49,168,169].

The literature further indicates that even after birth, the adrenal medulla remains immature, and its functions are likely carried out by the organs of Zuckerkandl, which reach maximal development by about three years of age. Thereafter, these organs undergo involution in parallel with the growth and maturation of the adrenal medulla [170].

Thus, N. A. Smitten’s theory regarding the rudimentary nature of carotid body cells gains substantial support when considered from the perspective of endocrine function. In evolutionary terms, beginning with cyclostomes and extending to mammals, the first tissue to undergo reduction is the chromaffin tissue of the branchial arteries. At the same time, the adrenal medulla is the most recent evolutionary development [44].

A similar phenomenon is observed in human ontogeny. Almost immediately after birth, the carotid body ceases to synthesize tyrosine hydroxylase. After the second year of postnatal life, the organs of Zuckerkandl undergo involution, and catecholamine production shifts to the adrenal medulla. Concurrently, in our view, the function of the carotid body transitions from an endocrine to a chemoreceptive role.

Thus, we can conclude that the carotid body and the adrenal medulla, sharing a common evolutionary origin, began to develop relatively independently from a certain point, specializing in different functions. It is this initial commonality and subsequent divergence in the evolution of chromaffin cells that are responsible for the molecular similarities and differences between the carotid body and the adrenal medulla, which are being identified by modern researchers [171].

### 6.3. A Proposed Model of Carotid Body Function

Taking into account both our findings and those of other investigators, it can be asserted that the carotid body indeed possesses an endocrine function at least at some stages of ontogenesis. From this perspective, a fundamentally new model of the organ’s operation emerges.

As part of the unified sympathoadrenal system, the carotid body is the phylogenetic homolog of the chromaffin cells of cyclostomes and fish. In these species, chromaffin cells receive no direct innervation and are relatively independent of the nervous system. They directly sense changes in the chemical composition of the incoming blood and, in response to various stimuli, release catecholamines. In the context of poorly developed neural regulation, this humoral pathway was the only possible mechanism [44].

Thus, from the very beginning of their evolutionary trajectory, chromaffin tissues carried out two functions simultaneously—sensory and endocrine.

As evolution progressed, the entire chromaffin system underwent significant reorganization. Amphibians illustrate this particularly well. In caecilians, the chromaffin tissue still displays a segmented organization corresponding to the segmentation of the adjacent blood vessels. In urodeles (salamanders), segmentation of the chromaffin tissue is lost. However, in anurans (frogs), along with the emergence of a more advanced segmented sympathetic nervous system, secondary metamerism of the chromaffin tissue appears, but now linked to the autonomic nervous system [44].

Further along the evolutionary line, chromaffin tissue became increasingly centralized within a single organ—the adrenal gland—with corresponding reduction in extra-adrenal chromaffin tissue. Centralization also occurred within the adrenal gland itself. In reptiles and birds, the adrenal medulla is composed of multiple scattered groups of chromaffin cells embedded within the cortex. In mammals, by contrast, these cells are consolidated into a single cluster beneath the cortex [44].

Thus, according to Smitten, with the evolutionary development of more complex nervous regulation, chromaffin tissue became progressively centralized, increasingly dependent on the nervous system. Extra-adrenal chromaffin tissue, deprived of activity, underwent atrophy and persisted in modern mammals only as a rudiment [44].

However, interestingly, chromaffin cells of the carotid body retain a degree of relative independence even in mammals. Following complete denervation, no degeneration of type I or type II cells occurs [72,98]. By contrast, cells initially dependent on innervation—for example, taste bud cells of the tongue—disappear after denervation, being replaced by ordinary epithelium [172,173,174].

Grigorieva shared a similar perspective with Smitten. In her view, the carotid body had no relation to chemosensory function, serving solely as an endocrine organ. She argued that the abundant innervation of vascular walls alone was sufficient to ensure chemoreflexes to changes in blood gas composition [43].

Nevertheless, although Smitten and Grigorieva were correct in highlighting the endocrine function, their rejection of the chemosensory role cannot be accepted. As shown in the literature review, in vitro experiments involving isolated stimulation of the carotid body demonstrated that the chemoreceptor response is localized precisely within this organ. Moreover, cryodestruction experiments convincingly demonstrated that sinus nerve chemosensitivity disappears, even though barosensitivity is restored over time.

As V. V. Yaglov and colleagues correctly observed, investigators were confounded by the idea that a single cell type might perform two distinct functions [175]. This is why neither Grigorieva nor Smitten fully developed their theories. Conversely, their opponents, focusing exclusively on the chemosensory role, failed to recognize the endocrine one.

**Figure 11:**
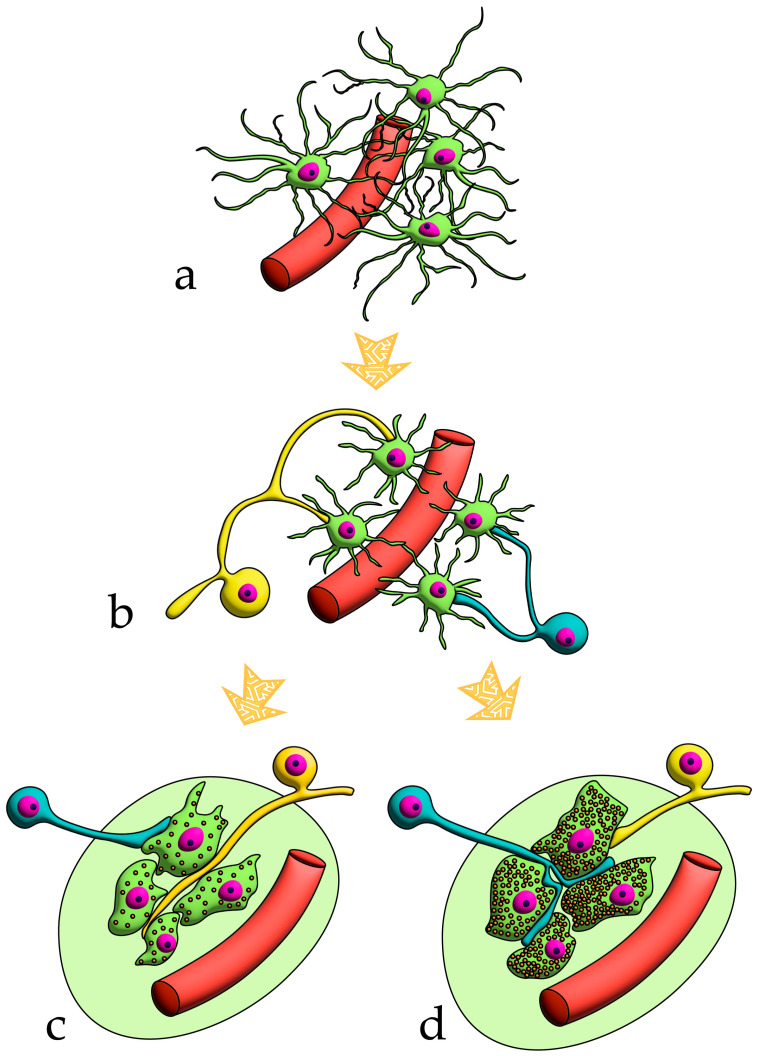
Proposed model of chromaffin tissue evolution. Afferent nerves are yellow; efferent nerves are turquoise. (**a**) Chromaffin cells, scattered near blood vessels, are largely uninnervated. They perform both a sensory function (detecting changes in the internal environment of the organism) and an effector (endocrine) function. (**b**) The cells become innervated: some of them receive predominantly sensory fibers, while others receive motor fibers. (**c**) Chromaffin cells specialize primarily in chemosensory function and form the carotid body. (**d**) Chromaffin cells specialize primarily in endocrine function and form the adrenal medulla.

Taking all of this into account, we may propose the following scheme of sympathoadrenal system evolution. Beginning with cyclostomes and fish, chromaffin tissue performed both chemosensory and endocrine functions. Over time, this tissue diverged into two groups: one specialized predominantly in efferent endocrine function, the other in chemosensory function (Figure 11).

Yet neither group lost the other role entirely; both retained them to a greater or lesser extent. For example, studies show that chromaffin cells of the adult adrenal medulla release catecholamines only in response to stimulation by sympathetic nerve fibers. However, after denervation, they regain the ability to release catecholamines in response to hypoxia [176,177]. Thus, beyond their primary function, they preserve chemosensitivity, which is normally suppressed by efferent sympathetic input.

The same can be said of the carotid body. Although its primary role in adults is to detect the partial pressures of blood gases, during the antenatal period it retains its endocrine function, compensating for the immaturity of the adrenal medulla.

The unity of the adrenal medulla and the carotid body is further supported by the fact that both respond to acute hypoxia with catecholamine release and to chronic hypoxia with hyperplasia of chromaffin cells [54,178]. Evidence that chronic hypoxia increases catecholamine synthesis in both organs reinforces the idea that endocrine function is intrinsic to both.

Perhaps the most intriguing aspect concerns the sensory innervation of the carotid body. In most studies, efferent and reciprocal synapses have been observed on endings belonging to axons of sensory neurons from the glossopharyngeal ganglion. Yet all investigators interpreted these synapses unidirectionally, as mechanisms regulating chemosensory function.

Experiments have shown that electrical stimulation of the glossopharyngeal nerve reduces the chemosensory response of type I cells, mediated by catecholamine release [179]. Explanations of this phenomenon solely in terms of chemosensory function are unconvincing.

In our view, this can be better explained by the existence of an axon reflex within the carotid body. According to Zavarzin, sensory neurons may also exert efferent functions. This explains, for instance, cutaneous-vascular reactions in response to irritation [180].

**Figure 12:**
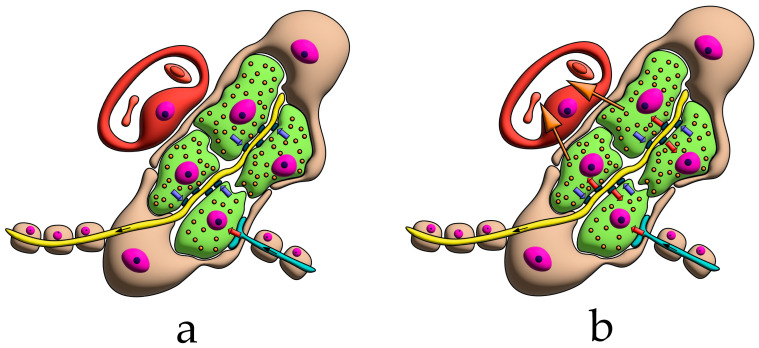
A proposed model of carotid body function. The afferent sinus nerve is yellow; the efferent sympathetic nerve is turquoise. (**a**) Minimal changes in blood gas composition. The cells perform a purely sensory function. Impulse transmission occurs solely from type I cells to the sinus nerve. (**b**) Marked changes in blood gas composition. Hyperactivity of type I cells activates reverse impulse transmission from the sinus nerve to them. A limited release of dopamine into the blood occurs. Pathway (**b**) is likely predominant during the antenatal period.

Something similar likely occurs in the carotid body. Minor changes in blood chemistry do not necessitate activation of the entire sympathoadrenal system. However, under severe hypoxia, type I cells must more strongly excite sinus nerve fibers by releasing larger amounts of acetylcholine and ATP as mediators. In response, these sensory fibers not only transmit signals to the brainstem but also, through their efferent synapses, act back on type I cells. This feedback amplifies catecholamine release—including dopamine (Figure 12). This may explain Hellström’s observation that dopamine and norepinephrine release from type I cells under hypoxia diminished if the sinus nerve had been transected 14 days earlier [108].

This hypothesis also neatly accounts for Zapata’s observation of dopamine’s biphasic effect: initial inhibition of sinus nerve activity, followed by excitation upon repeated fractional administration [111]. Presumably, initial stimulation by acetylcholine increases sinus nerve firing. If homeostasis is not restored, type I cells, both autonomously and under sinus nerve influence, begin to release dopamine in pulses. Dopamine then further enhances sinus nerve activity and likely enters the bloodstream, producing systemic effects. However, in adults these endocrine effects are relatively limited, since the carotid body’s endocrine role is largely reduced.

Thus, although type I cells partially lose their endocrine functions in adulthood, they retain them during embryonic and fetal life. This aligns with our findings of abundant tyrosine hydroxylase in embryonic and fetal cells, as well as Korkala’s detection of catecholamines themselves [161]. Hervonen likewise confirmed the presence of mature innervation of type I cells in the carotid body of fetuses during the second trimester [70].

It is well established that catecholamines significantly increase the survival of fetuses and newborns, particularly those experiencing hypoxia. This effect is attributed to both the direct influence of catecholamines on the cardiovascular system [181,182] and their role in stimulating pulmonary surfactant production by the fetal lungs [182]. Furthermore, catecholamines are also involved in the intrauterine regulation of glucose metabolism by modulating the function of pancreatic beta cells [183]. Thus, the presence of additional sources of catecholamine production aids in the more effective control of fetal and neonatal homeostasis during the period of adrenal medulla immaturity.

From this perspective, the coexistence of afferent, efferent, and reciprocal synapses becomes fully comprehensible. The remaining unresolved question is whether the sinus nerve can detect changes in blood chemistry without type I cells.

Initially, the answer appeared to be no, since cultured petrosal ganglion neurons showed no response to hypoxia [129], and carotid bodies subjected to cryodestruction exhibited no chemosensitivity even after reinnervation [128]. However, these studies can be criticized.

In the first case, neurons were deprived of their glial sheaths. It is now well established that glial cells do far more than provide mechanical support: they actively participate in neuronal metabolism and regulate ion exchange in the extracellular space [5]. Could glial cells and type II cells in the intact organism enhance the chemosensitivity of neurons themselves? Probably yes. Early studies had already shown that hypoxia and hypercapnia directly influence nerve fiber activity [184,185]. Still, it must be acknowledged that the response of nerve fibers alone is insufficient for a rapid and adequate reaction to hypoxia.

## 7. Conclusions

The centuries-long history of research on this small organ, located at the bifurcation of the carotid artery, demonstrates that the study of any organ cannot be conducted in isolation, without regard for the larger system and its role within it. A multicellular organism is an integrated system that always responds as a whole to external or internal influences, even if the changes observed by researchers appear to be localized in a single organ. Over millions of years of evolution, multicellular organisms have developed a vast array of mechanisms for maintaining homeostasis and adapting to the external environment, achieved through the coordinated interaction of different organs. Regardless of their embryonic origin, organs operate within unified functional systems. This fact alone demonstrates the impossibility of treating any type of systemic pathology through local intervention targeting only a single link in the chain. Thus, ideas of targeted therapy directed solely at modifying the activity of the carotid body are unfounded.

The carotid body is but one of many derivatives of the neural crest, which during development gives rise to numerous tissue structures. Some of these structures form a complex system controlling external respiration. From the outset, neural crest derivatives are closely interconnected, both among themselves and with other systems of the organism. This interaction involves multiple links through which signals about changes in the internal environment are transmitted to effector organs. Along this pathway, signals undergo significant transformation, while effector organs, via feedback mechanisms, in turn modify the activity of afferent organs.

In this work, we have presented a new model of the carotid body that integrates its endocrine and chemosensory features. We have also reinterpreted the carotid body as an essential component of a unified sympathoadrenal system, rather than a separate, isolated chemoreceptor. According to this model, the function of the carotid body in the antenatal period differs significantly from its function during postnatal ontogeny.

Our analysis of an extensive body of literature, together with our own studies, showed that carotid body cells exhibit high levels of tyrosine hydroxylase synthesis throughout prenatal development. At the earliest stages of embryogenesis, the morphology and immunohistochemical profile of the carotid body closely resemble those of the organs of Zuckerkandl. At the same time, the adrenal medulla consists only of small clusters of immature cells and is incapable of fulfilling its function. Thus, at the antenatal stage of ontogenesis, the carotid body, along with the organs of Zuckerkandl, likely performs an endocrine role, compensating for the insufficiency of catecholamine synthesis by the immature adrenal medulla.

In the postnatal period, the endocrine function of the carotid body is largely reduced, and the organ primarily serves as a peripheral chemosensor. However, some structural features indicative of its prior role (e.g., efferent synapses, lack of cell polarity, etc.) are preserved. It is the persistence of these structural features into the postnatal period that has long been a point of contention for researchers who disputed its chemosensory function.

This controversial duality of the carotid body only becomes clearer when researchers shift from an isolated view of the organ to an integrative consideration of it within the entire system. Only a comprehensive study of the organism as a whole will provide the necessary basis for the correct interpretation of observed findings—an essential prerequisite for developing new, effective approaches to treating various pathologies. For this reason, even in the modern era of single-cell studies, as researchers delve even deeper into the mysteries of molecular interactions, classical methods of comparative histology and anatomy remain highly relevant and constitute an indispensable complement to molecular research.

## Figures and Tables

**Figure 2:**
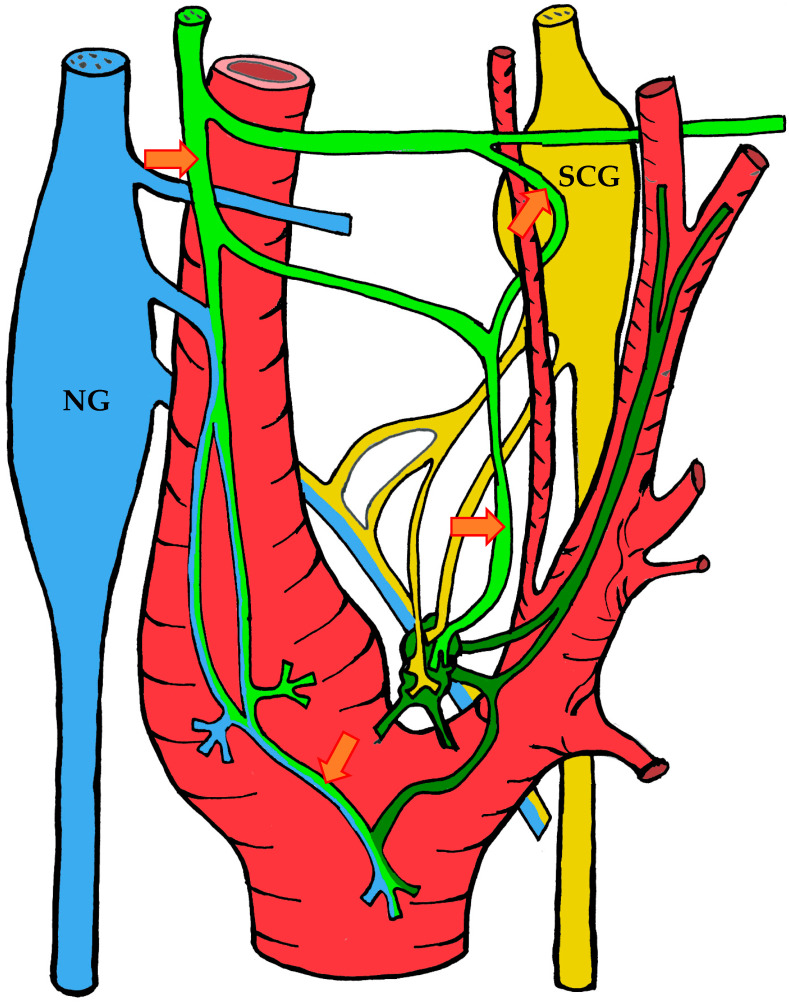
The course of nerve fibers innervating the carotid body according to Sheehan and Mulholland [68]. The vagus nerve and its branches are shown in blue, the glossopharyngeal nerve and its branches in lime green, and sympathetic nerve fibers in yellow. NG—inferior ganglion of the vagus nerve (nodose ganglion), SCG—superior cervical ganglion, arrow indicates the sinus nerve and its branches.

**Figure 3:**
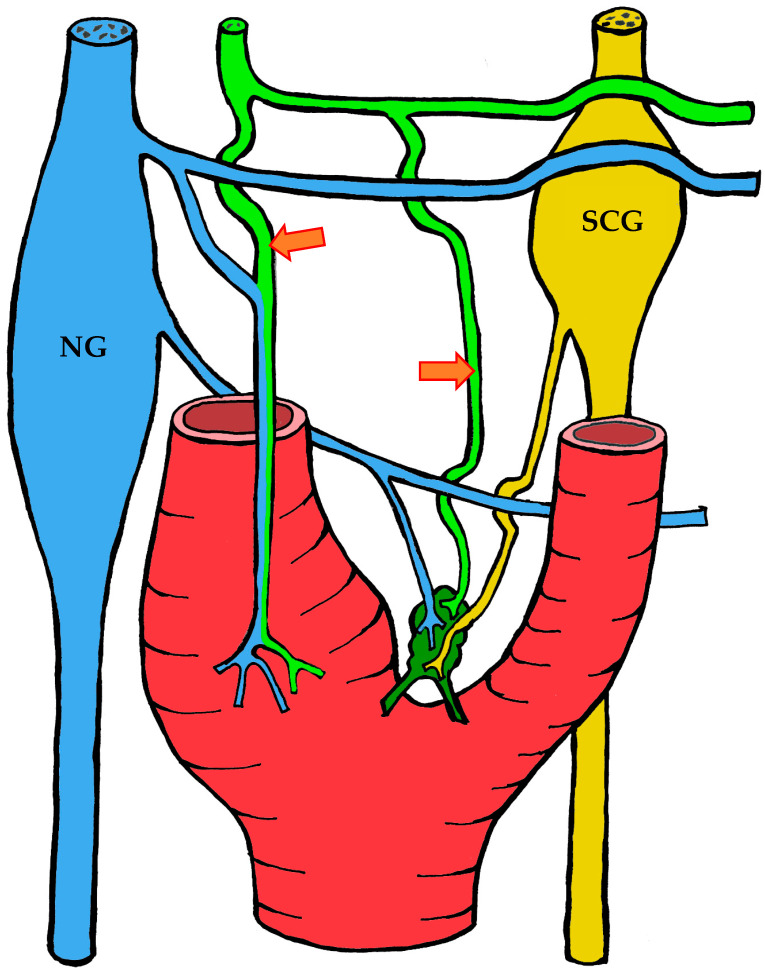
The course of nerve fibers innervating the carotid body according to Sheehan and Mulholland [68]. The vagus nerve and its branches are shown in blue, the glossopharyngeal nerve and its branches in lime green, and sympathetic nerve fibers in yellow. NG—inferior ganglion of the vagus nerve (nodose ganglion), SCG—superior cervical ganglion, arrow indicates the sinus nerve and its branches.

**Figure 4:**
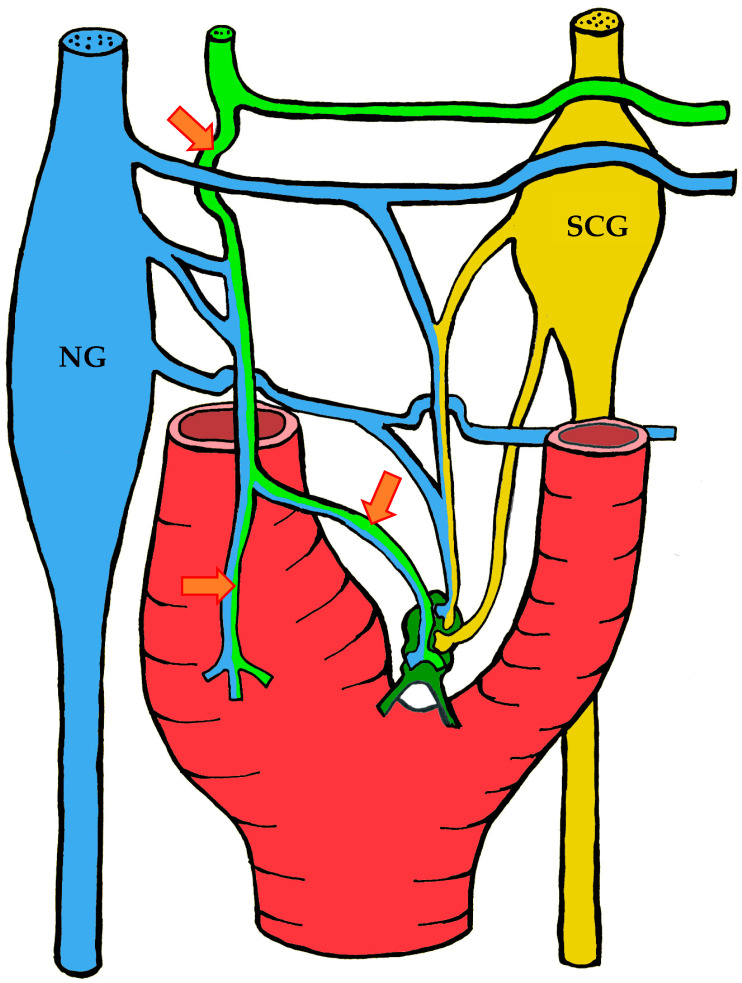
The course of nerve fibers innervating the carotid body according to Sheehan and Mulholland [68]. The vagus nerve and its branches are shown in blue, the glossopharyngeal nerve and its branches in lime green, and sympathetic nerve fibers in yellow. NG—inferior ganglion of the vagus nerve (nodose ganglion), SCG—superior cervical ganglion, arrow indicates the sinus nerve and its branches.

**Figure 5:**
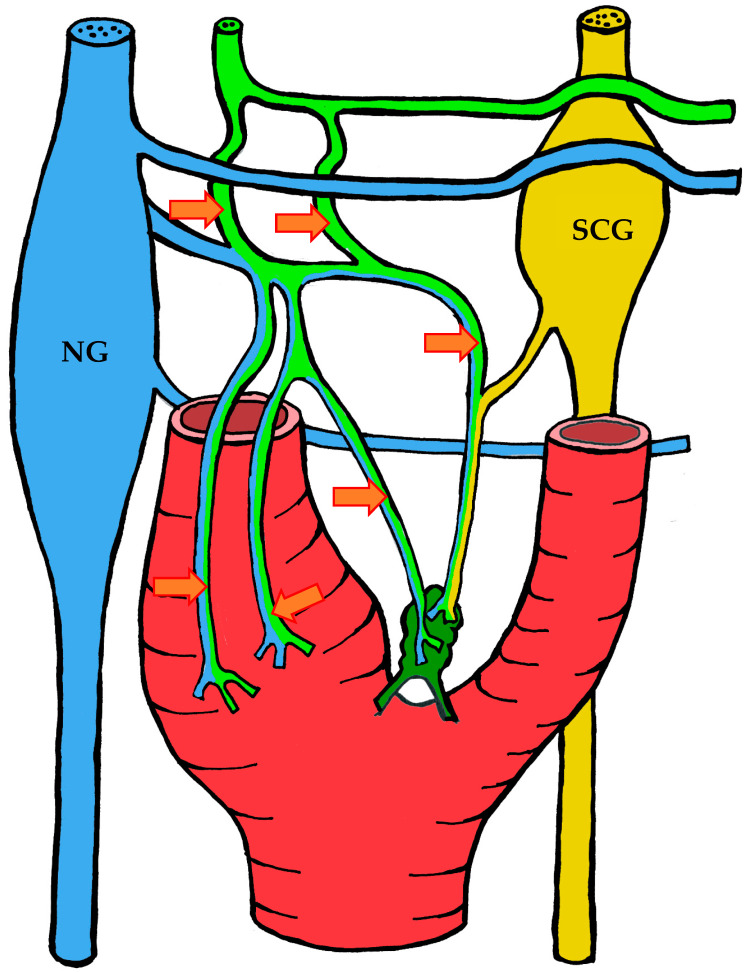
The course of nerve fibers innervating the carotid body according to Sheehan and Mulholland [68]. The vagus nerve and its branches are shown in blue, the glossopharyngeal nerve and its branches in lime green, and sympathetic nerve fibers in yellow. NG—inferior ganglion of the vagus nerve (nodose ganglion), SCG—superior cervical ganglion, arrow indicates the sinus nerve and its branches.

**Figure 6:**
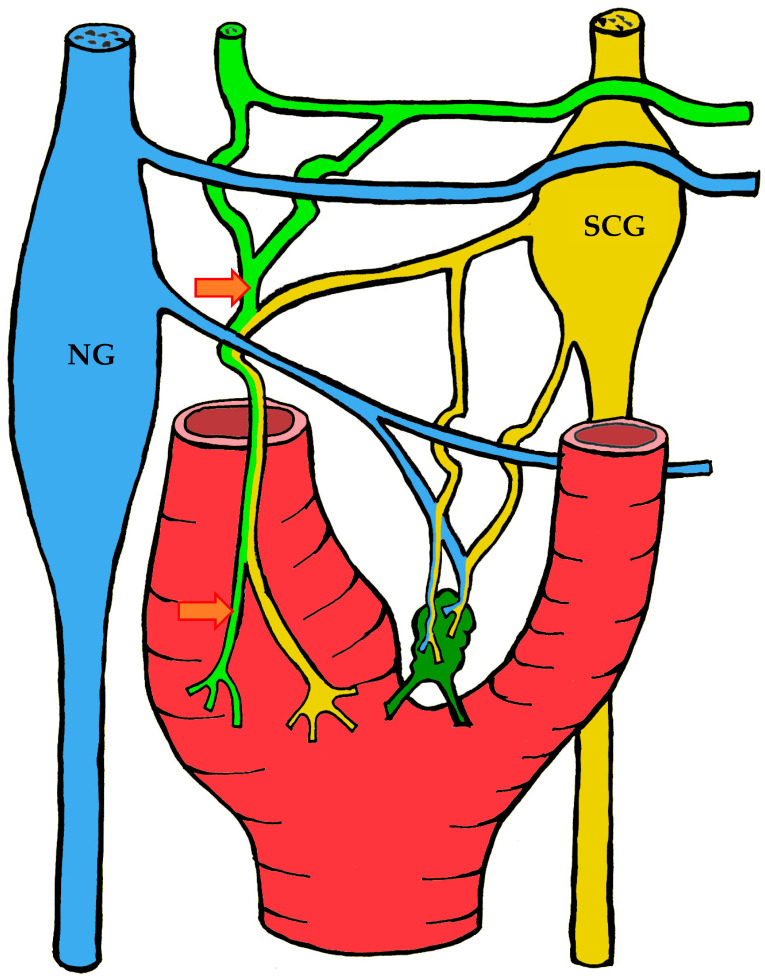
The course of nerve fibers innervating the carotid body according to Sheehan and Mulholland [68]. The vagus nerve and its branches are shown in blue, the glossopharyngeal nerve and its branches in lime green, and sympathetic nerve fibers in yellow. NG—inferior ganglion of the vagus nerve (nodose ganglion), SCG—superior cervical ganglion, arrow indicates the sinus nerve and its branches.

**Figure 7:**
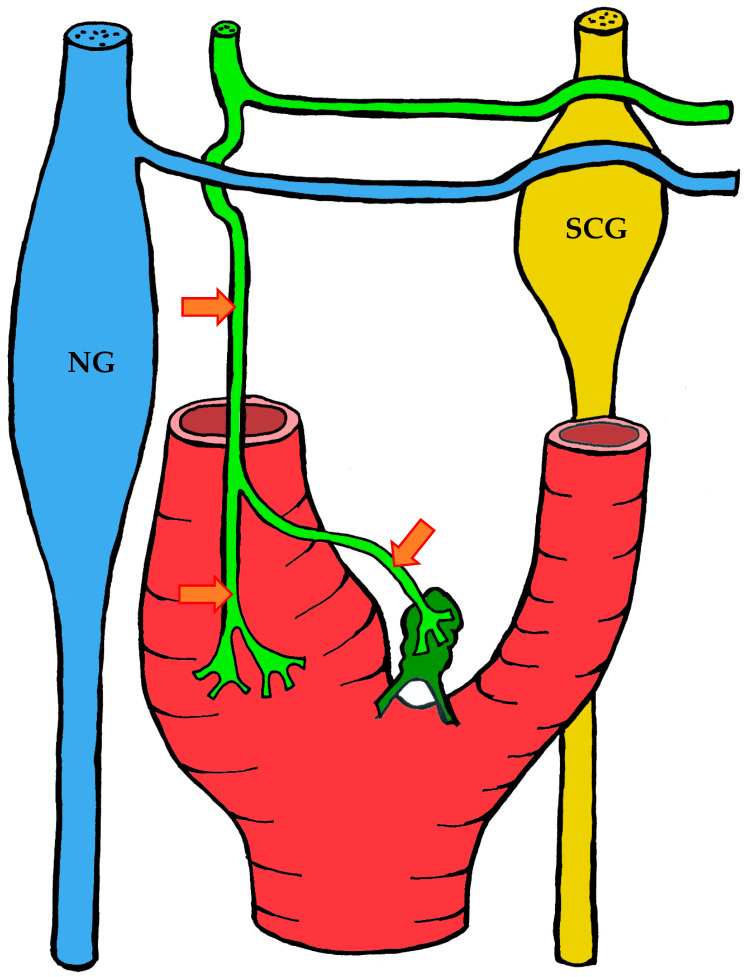
The course of nerve fibers innervating the carotid body according to Toorop [66]. The vagus nerve and its branches are shown in blue, the glossopharyngeal nerve and its branches in lime green, and sympathetic nerve fibers in yellow. NG—inferior ganglion of the vagus nerve (nodose ganglion), SCG—superior cervical ganglion, arrow indicates the sinus nerve and its branches.

**Figure 8:**
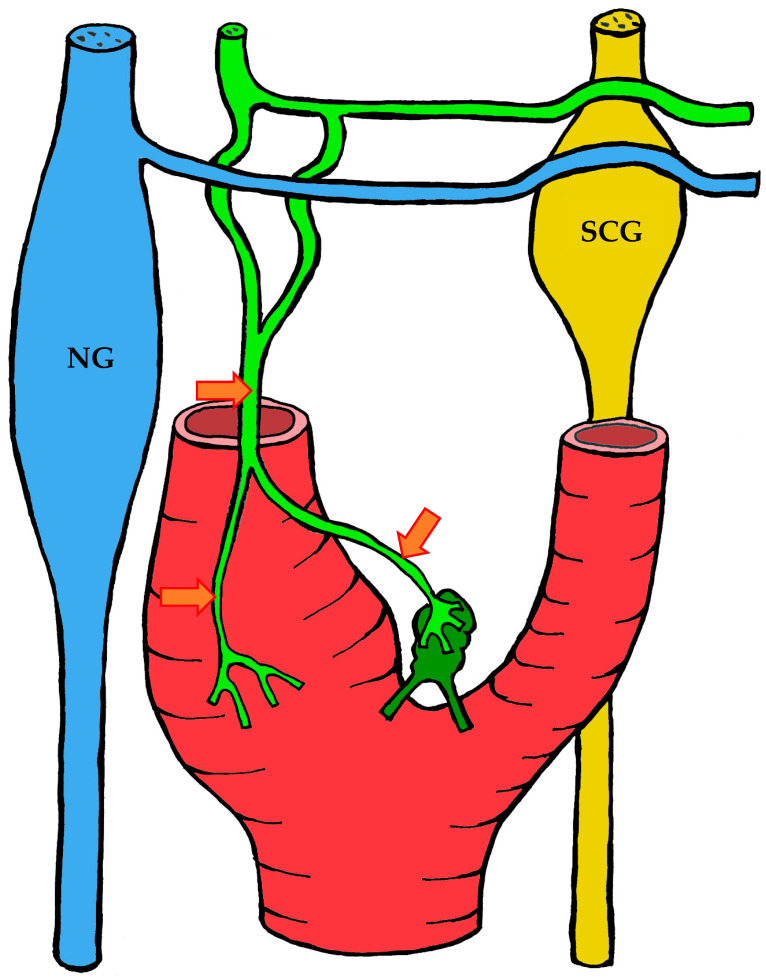
The course of nerve fibers innervating the carotid body according to Toorop [66]. The vagus nerve and its branches are shown in blue, the glossopharyngeal nerve and its branches in lime green, and sympathetic nerve fibers in yellow. NG—inferior ganglion of the vagus nerve (nodose ganglion), SCG—superior cervical ganglion, arrow indicates the sinus nerve and its branches.

**Figure 9:**
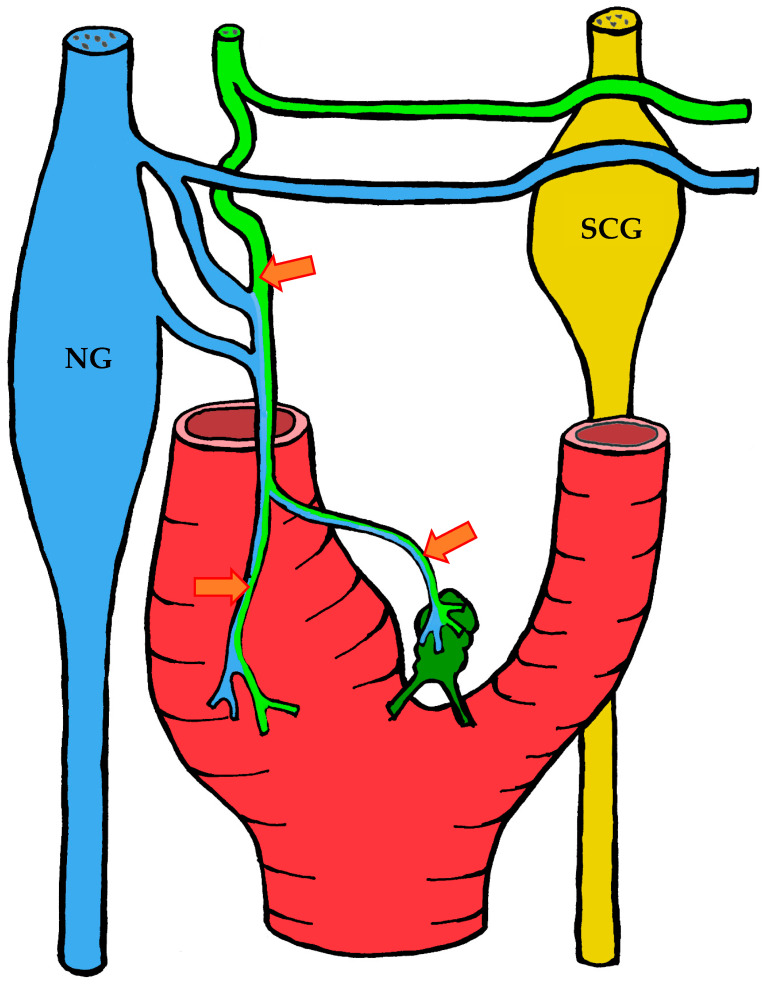
The course of nerve fibers innervating the carotid body according to Toorop [66]. The vagus nerve and its branches are shown in blue, the glossopharyngeal nerve and its branches in lime green, and sympathetic nerve fibers in yellow. NG—inferior ganglion of the vagus nerve (nodose ganglion), SCG—superior cervical ganglion, arrow indicates the sinus nerve and its branches.

**Figure 10:**
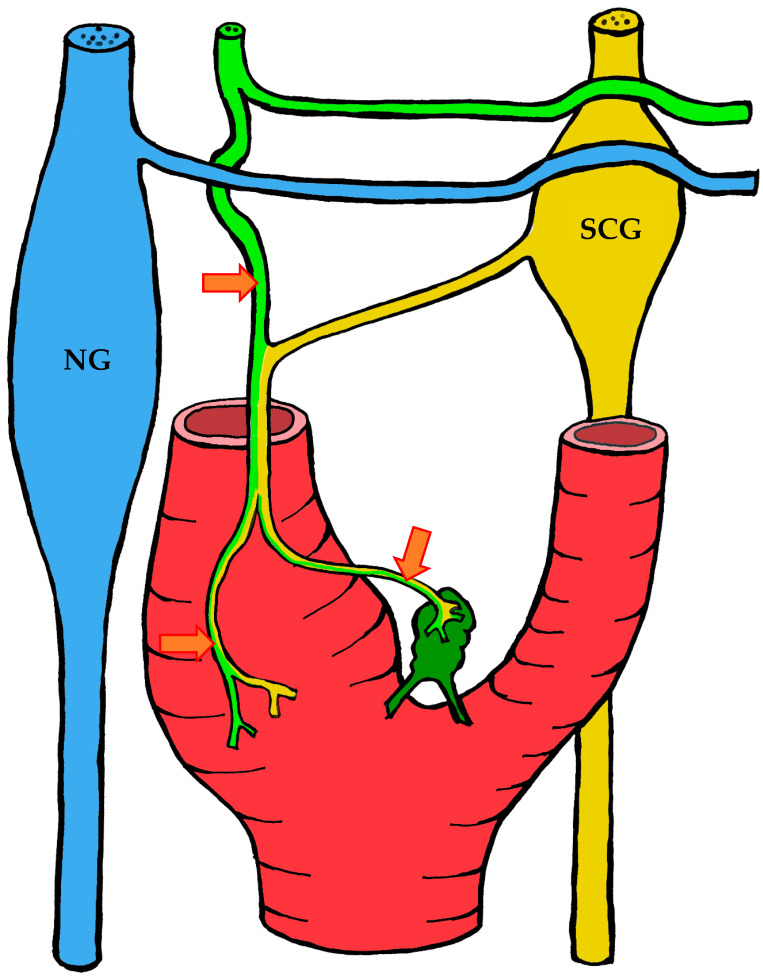
The course of nerve fibers innervating the carotid body according to Toorop [66]. The vagus nerve and its branches are shown in blue, the glossopharyngeal nerve and its branches in lime green, and sympathetic nerve fibers in yellow. NG—inferior ganglion of the vagus nerve (nodose ganglion), SCG—superior cervical ganglion, arrow indicates the sinus nerve and its branches.

**Table 1:** Frequency of carotid body locations according to Khan et al. [51] and Smith et al. [52] (500 arteries from 250 individuals). Values given in percentages.

Location	Right	Left	Both Sides
Carotid bifurcation	87.2	87.2	87.2
External carotid artery	5.6	6.4	6.0
Internal carotid artery	5.2	3.6	4.4
Ascending pharyngeal artery	1.6	2.4	2.0
Common carotid artery	0.4	0.4	0.4

**Table 2:** Frequency of carotid body shapes according to Khan et al. [51] and Smith et al. [52] (500 arteries from 250 individuals). Values given in percentages.

Shape	Right	Left	Both Sides
Single ovoid	90.0	90.4	90.2
Double ovoid	5.2	3.6	4.4
Bilobed	4.4	5.6	5.0
Leaf-like	0.4	0.4	0.4

## Data Availability

The original contributions presented in this study are included in the article. Further inquiries can be directed to the corresponding author.

## Abbreviations

The following abbreviations are used in this manuscript:

TH	Tyrosine hydroxylase
βIII	βIII-tubulin
GABA	gamma-aminobutyric acid
PGP9.5	ubiquitin carboxy-terminal hydrolase L1
TASK-1	potassium two pore domain channel subfamily K
ATP	Adenosine triphosphate
ER	endoplasmic reticulum
SIDS	Sudden Infant Death Syndrome
